# Characterization of a rhabdomyosarcoma reveals a critical role for SMG7 in cancer cell viability and tumor growth

**DOI:** 10.1038/s41598-023-36568-5

**Published:** 2023-06-22

**Authors:** Alexander J. Steiner, Yang Zheng, Yi Tang

**Affiliations:** Department of Regenerative and Cancer Cell Biology, Albany Medical College, 47 New Scotland Avenue, Albany, NY 12208 USA

**Keywords:** Cancer, Cell biology

## Abstract

Soft-tissue sarcomas (STSs) are a rare and diverse group of mesenchymal cancers plagued with aggression, poor response to systemic therapy, and high rates of recurrence. Although STSs generally have low mutational burdens, the most commonly mutated genes are tumor suppressors, which frequently acquire mutations inducing nonsense-mediated mRNA decay (NMD). This suggests that STS cells may exploit NMD to suppress these anti-cancer genes. To examine the role that the NMD factor SMG7 plays in STS, we developed an inducible knockout mouse model in the *Trp53*^*−/−*^ background. Here, we isolated a subcutaneous STS and identified it as a rhabdomyosarcoma (RMS). We report that knockout of SMG7 significantly inhibited NMD in our RMS cells, which led to the induction of NMD targets GADD45b and the tumor suppressor GAS5. The loss of NMD and upregulation of these anti-cancer genes were concomitant with the loss of RMS cell viability and inhibited tumor growth. Importantly, SMG7 was dispensable for homeostasis in our mouse embryonic fibroblasts and adult mice. Overall, our data show that the loss of SMG7 induces a strong anti-cancer effect both in vitro and in vivo. We present here the first evidence that disrupting SMG7 function may be tolerable and provide a therapeutic benefit for STS treatment.

## Introduction

Soft-tissue sarcomas (STSs) are aggressive cancers that respond poorly to chemotherapy and have few efficacious options for targeted therapy^1^. STSs are mesenchymal cancers that derive from several different lineages, such as cartilage, adipose, fibrous, vasculature, smooth muscle, and skeletal muscle^2^. STSs are further categorized by their histology, giving rise to more than 70 histological subtypes^3^. STSs in the adult population are rare (~ 1%), with the incredible diversity of STS resulting in several subtypes being exceedingly rare. The rarity of STS hampers both the exploration of targeted therapies and guidance for systemic therapies. The treatment of STS is further complicated due to the heterogeneous response to systemic therapies across subtypes, such as in rhabdomyosarcoma (RMS). RMS is a skeletal muscle sarcoma characterized by nuclear staining of the myogenic regulatory factors MyoD1 and/or myogenin^4,5^. The four main histological subtypes of RMS are embryonal, alveolar, spindle cell/sclerosing, and pleomorphic^5^. RMS is a main cancer seen in children, with subtypes mostly presenting as embryonal or alveolar RMS^6,7^. The high incidence of RMS in the paediatric population has allowed the standardization of treatment using a multimodal chemotherapeutic strategy, resulting in a 5-year overall survival (OS) rate of 61%^6^. Pleomorphic RMS is mainly an adult subtype of RMS that responds poorly to the same paediatric multimodal treatment, resulting in a 5-year OS rate of 30%^5,8^. The poor response rate of chemotherapeutics seen in pleomorphic RMS is common across STSs, which highlights the need for the discovery of viable therapeutic targets.

As STSs exhibit low somatic mutation burdens, with the most frequently mutated genes being tumor suppressors, it may be beneficial to search for therapy targets in the cancer fitness gene category rather than the driver oncogene category^9^. Cancer fitness genes are distinct from driver oncogenes as they are dispensable for initial transformation^10^. These genes are utilized by cancer cells to gain proliferative, survival, stress-response, or metastatic benefits during cancer progression, and they tend to be unnecessary for somatic cells under homeostatic conditions^10^. Nonsense-mediated mRNA decay (NMD) is a pathway that consists of numerous factors, some of which may fall into the cancer fitness gene category^11,12^.

NMD is a quality control and transcriptomic regulatory pathway largely targeting mRNAs that harbor premature termination codons (PTCs), upstream open reading frames (uORFs) where the termination codon is interpreted as a PTC, or unusually long (> 1 kb) 3′ untranslated regions (UTRs)^13,14^. NMD is activated when the helicase/ATPase UPF1 is phosphorylated by the serine/threonine kinase SMG1. The complex formation of UPF1, UPF2 and UPF3b further stimulates UPF1 activity^15^. Phosphorylation of UPF1 creates binding sites for the SMG5/SMG7 heterodimer^16^. SMG5/7 bind to UPF1 and recruit a deadenylation complex, a decapping complex, and the endonuclease SMG6, which leads to the complete degradation of the targeted mRNA^17–19^. SMG5/7 also recruit the phosphatase PP2A to dephosphorylate and recycle UPF1, thus allowing NMD to begin on a new target^17,20^.

STS cells may utilize NMD to give themselves a proliferative and survival advantage. A plethora of NMD targets have been identified, with several being linked to apoptotic and cell cycle arrest effects, such as GADD45b and the tumor suppressor lncRNA GAS5^21–23^. While GAS5 is downregulated in numerous cancers, including osteosarcoma, the role that GADD45b plays in cancer is less clear, as there are reports of decreased expression of GADD45b in some cancers and increased expression in other cancers^24,25^. Cancer cells may also exploit NMD to decrease the amount of neoantigen production to avoid a strong immune microenvironment^26^. Although, this may not be as relative to STSs as they generally have low mutation burdens, with the most frequently mutated genes being the tumor suppressors RB1, ATRX, and TP53^9,27,28^. The same tumor suppressors frequently acquire NMD-eliciting mutations in STSs, which suggests that NMD may provide STS cells with a fitness advantage by downregulating tumor suppressors^29^. The relationship between mutated genes in STS and NMD-targeted genes suggests that NMD may be a pathway worthy of exploration for therapeutic targets in STSs.

We and others have shown that SMG7 plays key roles in the p53-mediated DNA damage response and in NMD, but its role in STS is unexplored^30–33^. The role that p53 plays in cancer has been heavily studied, with mutations and deep deletions being common in several subtypes of STS, including RMS^9,34–36^. The role that SMG7 and other NMD factors may play in cancer is a relatively novel and burgeoning field. There is evidence to suggest that NMD can exhibit two dichotomous paradigms in cancer, pro- or anti-tumorigenic roles^11,12,37–40^. NMD may act in a pro-tumorigenic role by downregulating tumor suppressors, pro-inflammatory neoantigens, and desensitizing cancer cells to chemotherapeutics^41–44^. SMG7 and several other NMD factors are upregulated in a subgroup of colorectal cancers (CRC) with microsatellite instability (MSI)^44^. Inhibition of NMD in these cells led to the expression of hundreds of PTC-containing mRNAs with a concomitant decrease in the proliferation of MSI CRC cells, both in vitro and in vivo^44^. NMD may also act in an anti-tumorigenic role by suppressing stress-induced factors, epithelial-mesenchymal transition, invasion, migration, metastasis, and by sensitizing cells to cytokine-induced death^45–48^. The knockdown of SMG7 in breast cancer cells decreased the effectiveness of TNFα-mediated cell death^47^. TNFα and melphalan are utilized in isolated limb perfusion for the treatment of extremity STSs, suggesting that targeting SMG7 function could have negative effects in this route of treatment^49–51^. These combined studies intimate that targeting SMG7 may provide either beneficial or detrimental effects in STS treatment.

In this study, we utilize a genetically engineered mouse model, which has the ability to conditionally knock out SMG7 and generate spontaneous STSs, to isolate a spontaneous RMS and analyze what role SMG7 plays in this STS subtype. We show that the loss of SMG7 leads to decreased proliferation and increased apoptosis in our RMS cells, both in vitro and in vivo. We also show that short-term knockout of SMG7 is tolerable in adult mice, with few gross anatomical changes in organ sizes. Our data suggests that targeting SMG7 function may be tolerable and provide a therapeutic benefit for STS treatment.

## Results

The two main models used to study STS are patient-derived xenografts (PDXs) and genetically engineered mouse models (GEMMs), both of which have their own advantages and disadvantages^52^. We elected to go with a GEMM as we are interested in the role of SMG7 in both tumorigenesis and the tumor microenvironment. Several NMD factors have been shown to be required for embryogenesis, including SMG7 in *Arabidopsis thaliana* and *Danio rerio*^53–59^. Given this, we utilized the knockout-first design to create a *Smg7* conditional knockout mouse (*Smg7*^*flox/flox*^) in the C57BL/6 background (see Supplemental Fig. S1 online)^60,61^. To induce diverse de novo tumorigenesis, we made use of the archetypal *Trp53*^*−/−*^ (also known as *p53*^*−/−*^) genotype, whereby STSs and lymphomas form in *p53*^*−/−*^ mice at a high frequency and within six months^62,63^. The primary tumor cells derived from our syngeneic model can then be introduced into fully immunocompetent mice. The ability to generate several, unique STSs provides additional benefit as STSs are a very heterogeneous group of cancers, and SMG7 may play different roles in different histological subtypes. Generating *p53*^*−/−*^ sarcomatous tumors is also germane, as the MDM2-p53 pathway is one of the most frequently mutated pathways in STS, with deep deletions of p53 being common in leiomyosarcoma, undifferentiated pleomorphic sarcoma, and myxofibrosarcoma^9^.

### Establishing and Characterizing STS Cell Lines

**Figure 1:**
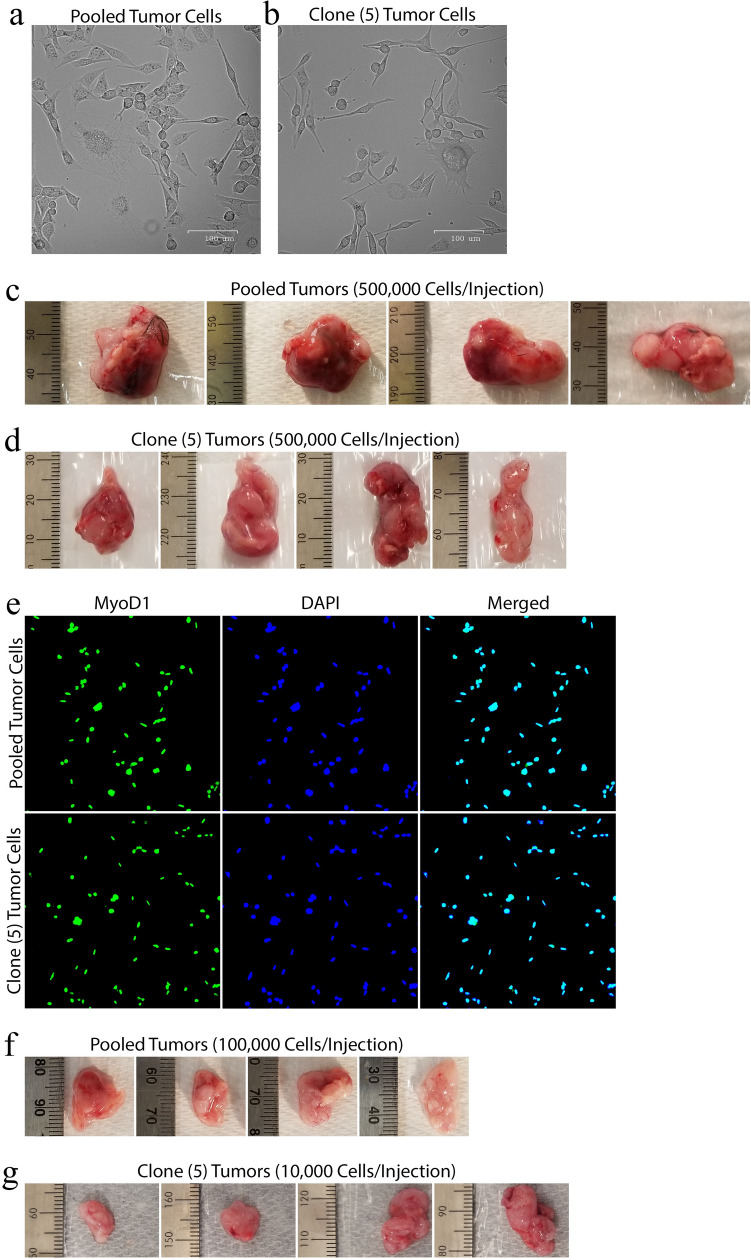
Establishing and Characterizing Primary STS Cell Lines. (**a** and **b**) Phase-contrast images (×100) of tumor (**a**) and clone 5 (**b**) populations (scale bars = 100 μm). (**c** and **d**) 500,000 tumor (**c**) and clone 5 (**d**) cells were subcutaneously injected into the flanks mice and allowed to grow for 8 days before mice were sacrificed and tumors were collected. (**e**) Representative immunofluorescence images of tumor and clone 5 cells processed and stained for MyoD1 (green) and counterstained with DAPI (blue). (**f**) 100,000 tumor cells were injected subcutaneously into the flanks of mice and allowed to grow for 9 days before mice were sacrificed and tumors were collected. (**g**) 10,000 clone 5 cells were subcutaneously injected into the flanks of mice and allowed to grow for 14 days before mice were sacrificed and tumors were collected. ([**c**,**d**,**f**, and **g**] N = 4 mice).

Our genetically engineered mice generated several tumors. As predicted in the literature, most of the spontaneous tumors were lymphomas^62,63^. We were able to isolate several sarcomatous tumors (8 tumors total). From one of these tumors, we established a primary tumor cell line that exhibited the ability to form tumors with 100% penetrance after subcutaneously injecting the tumor cells into the flanks of our mice (4 of 4 injected-mice developed large tumors). For the remainder of isolated tumors, we encountered issues with tumor cells either failing to grow in vitro or failing to form tumors after injecting the cells subcutaneously into the flanks of our mice (4 mice used for each cell line injection). We were able to isolate and establish a STS cell line from a male *Smg7*^*flox/flox*^*; p53*^*−/−*^ mouse. This mouse generated a solid tumor located subcutaneously on the right-dorsal-caudal position (see Supplemental Fig. S1). We excised the tumor and established a primary cell line from the original tumor population. As sarcomatous tumors are inherently heterogeneous, we also used a limiting dilution method to establish several clonal cell lines from the original tumor population (see Supplemental Fig. S1). We have utilized the original tumor population and several clonal cell lines to corroborate our results. Our tumor cells were adherent and exhibited a pleomorphic phenotype that consisted of spindle-like, histiocyte-like, and large syncytia-like morphologies (see Fig. 1a). Individual clones displayed the same morphologies (see Fig. 1b and Supplemental Fig. S1). The tumor cells demonstrated a complete loss of contact inhibition and were able to proliferate unhindered for over 30 passages. To test if our primary cells retained their tumor-initiating capacity, we orthotopically introduced 500,000 of the original tumor cells and a clonal cell line into the subcutaneous flanks of our immunocompetent C57BL/6 mice. Eight days after injection, the mice were sacrificed and their tumors were collected. Both tumor and clonal cells formed large tumors with 100% penetrance (see Fig. 1c,d). We fixed the tumors and then stained them with haematoxylin and eosin (H&E). The H&E staining showed that the tumors were contained within a fibrous pseudocapsule and consisted of spindle-like and pleomorphic cells forming a storiform pattern (see Supplemental Fig. S1). The pleomorphic tumor cells had an abundance of dense, eosinophilic cytoplasm. Many cells exhibited mitotic figures with few having atypical mitotic figures. Several cells displayed nuclear atypia, especially the large, syncytia-like cells dotted throughout the tumor. None of the tumors exhibited anaplasia. Histologically, the tumor presented similarly to a high grade, spindle cell, pleomorphic rhabdomyosarcoma^5^. To test our hypothesis that our primary cell line was of myogenic lineage, we stained the tumor and clonal cells with MyoD1. Every observed tumor cell showed a strong nuclear MyoD1 staining, which is indicative of a rhabdomyosarcoma (RMS) (see Fig. 1e)^4,5^. To better gauge the tumor-initiating capacity of our RMS cell line, we injected four mice subcutaneously with 100,000 tumor and 10,000 clonal cells. All injected mice, regardless of low cell count, formed tumors with 100% penetrance (see Fig. 1f,g). These data suggest we have isolated a pleomorphic RMS with a robust tumor-initiating capacity.

### SMG7 is essential for RMS cell viability

**Figure 2:**
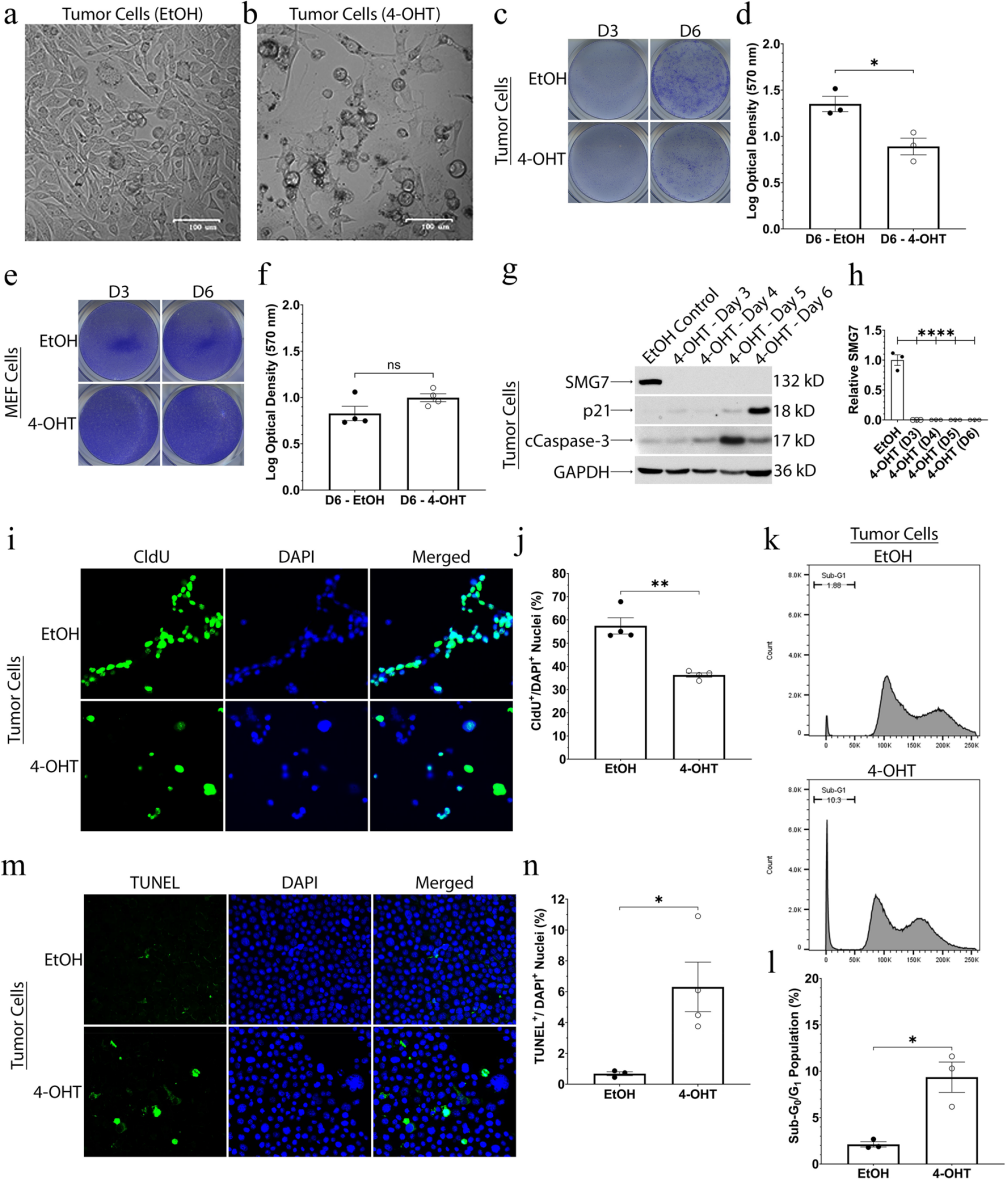
Loss of SMG7 inhibits proliferation and increases apoptosis in RMS cells. (**a** and **b**) Phase-contrast images (×100) of ethanol control (EtOH) (**a**) and 4-hydroxytamoxifen (4-OHT) (**b**) treated tumor cells 7 days after treatment. (**c** and **d**) Representative images of crystal violet staining of EtOH/4-OHT treated tumor cells on day 3 and day 6 are shown in (**c**). Optical densities of multiple, independent experiments of day 6 are quantified in (**d**). (**e** and **f**) Representative images of crystal violet staining of EtOH/4-OHT treated MEF cells on day 3 and day 6 are shown in (**e**). Optical densities of multiple, independent experiments of day 6 are quantified in (**f**). (**g** and **h**) Western blot of EtOH/4-OHT treated tumor cells showing progressive days of 4-OHT treatment. Cropped blots are shown and the original blots are available at the end of the Supplemental Figures. Quantification of SMG7 expression, relative to EtOH controls, from multiple experiments is shown in (**h**). (**i** and **j**) Representative immunofluorescence images of day 6 EtOH/4-OHT treated tumor cells that were processed and stained for CldU (green) and counterstained with DAPI (blue) are shown in (**i**). The percent of CldU^+^/DAPI^+^ nuclei from multiple, independent experiments are quantified in (**j**). (**k** and **l**) Representative flow cytometry plots of propidium iodide (PI) stained day 6 EtOH/4-OHT treated tumor cells with quantification of the sub-G_0_/G_1_ peak are shown in (**k**). The sub-G_0_/G_1_ population of multiple, independent experiments are quantified in (**l**). 200,000 singlet cells were analyzed in all flow experiments. (**m** and **n**) Representative immunofluorescence images of day 6 EtOH/4-OHT treated tumor cells that were processed and stained with TUNEL (green) and counterstained with DAPI (blue) are shown in (**m**). The percent of TUNEL^+^/DAPI^+^ nuclei from multiple, independent experiments are quantified in (**n**). ([**c**–**f** and **i**–**n**] Unpaired 2-tailed t-tests with means ± standard error of the mean (SEM) are provided in quantifications; [**g** and **h**] A one-way ANOVA utilizing the Dunnett correction with means ± SEM are provided in quantifications; N ≥ 3 ; *P < 0.05 ; **P < 0.01 ; ****P < 0.0001 ; ns = *P* > 0.05).

Knocking down certain NMD factors, such as UPF1 in MSI CRC cells, has resulted in a significant decrease of cell proliferation in vitro^44^. Antithetically, knockdown of SMG7 in breast cancer cells had no effect on viability and even sensitized the cells to TNFα-induced death^47^. These combined studies show that NMD factors may play opposing roles in different cancers. The role of SMG7 in STS is thus far unexplored. To test the effects of SMG7 loss on our RMS cells, we induced SMG7 knockout by treating our tumor and clonal cells with 4-hydroxytamoxifen (4-OHT), or ethanol (EtOH) as a vehicle control, and observed the effects. Over a period of five to seven days post-treatment, several of the tumor and clonal cells exhibited a significant shift in their usual, pleomorphic morphology to a rounded shape with thin cellular extensions adhering to the plate (see Fig. 2a,b and Supplemental Fig. S2). To test changes in cell viability after the loss of SMG7, we utilized the crystal violet assay. Three days after EtOH or 4-OHT treatment, the tumor (EtOH 0.6166 ± 0.01547 & 4-OHT 0.6702 ± 0.01166; *P* = 0.0505), clone 2 (EtOH 0.9891 ± 0.1754 & 4-OHT 0.8259 ± 0.1619; *P* = 0.53), clone 5 (EtOH 0.5677 ± 0.09193 & 4-OHT 0.5297 ± 0.05427; *P* = 0.73), and clone 8 (EtOH 1.186 ± 0.1734 & 4-OHT 0.8307 ± 0.1101; *P* = 0.12) populations did not exhibit a significant difference in cell viability. By day six, however, the tumor (EtOH 1.351 ± 0.08294 & 4-OHT 0.8913 ± 0.09021; *P* = 0.0199), clone 2 (EtOH 1.399 ± 0.07124 & 4-OHT 0.9741 ± 0.07305; *P* = 0.0141), clone 5 (EtOH 0.9156 ± 0.03248 & 4-OHT 0.4876 ± 0.06810; *P* = 0.0040), and clone 8 (EtOH 1.570 ± 0.1431 & 4-OHT 0.5656 ± 0.01831; *P* = 0.0022) populations all exhibited a significant decrease in cell viability following 4-OHT treatment (see Fig. 2c,d and Supplemental Fig. S3). If SMG7 falls into the cancer fitness gene category, then it would, ideally, be exploited by cancer cells but be dispensable for non-transformed cells under homeostatic conditions^10^. To test if SMG7 falls into the cancer fitness gene category, we isolated mouse embryonic fibroblasts (MEFs) from our *Smg7*^*flox/flox*^*; p53*^*−/−*^ mice. MEFs are mesenchymal, non-tumorigenic cells that also have multipotent stem-like properties^64–66^. These characteristics make MEF cells an ideal control, especially as NMD factors have been shown to be important for homeostatic maintenance of stem cells during embryogenesis and in developing and adult mice^67–70^. We treated our MEF cells with 4-OHT to induce the knockout of SMG7 and assessed changes in their viability using the crystal violet assay. The MEF cells had no significant change in cell viability on day 3 (EtOH 0.7291 ± 0.06379 & 4-OHT 0.8781 ± 0.1269; *P* = 0.3534) or day 6 (EtOH 0.8273 ± 0.07769 & 4-OHT 0.9976 ± 0.04261; *P* = 0.1029) (see Fig. 2e,f). These data suggest that SMG7 is dispensable for homeostasis and proliferation in our MEF cells, but is required for viability in our tumor and clonal cells.

### SMG7 is required for full RMS proliferative and survival capacity

The downregulation of the NMD factor UPF1 was shown to decrease proliferation in endometrial cancer and colorectal cancer cells^44,69^. Concomitantly, the endometrial cancer cells exhibited increased apoptosis^69^. A decrease in proliferation or an increase in apoptosis may explain the inhibited cell viability we see in our RMS cells following the loss of SMG7. To test if our cells expressed indicative markers of cell cycle arrest and apoptosis, we performed qPCR and western blot analysis of our tumor, clonal, and MEF cells. We analyzed the mRNA and protein expression of the cyclin-dependent kinase inhibitor p21 (also known as *Cdkn1a*) in our RMS cells on day 6. The loss of SMG7 induced a significant increase in the mRNA level of p21 in our tumor (ΔCt—EtOH 5.407 ± 0.2466 & 4-OHT 4.329 ± 0.3210; *P* = 0.0374) and clone 5 (ΔCt—EtOH 6.239 ± 0.4141 & 4-OHT 4.986 ± 0.2477; *P* = 0.0408) cells, as well as a clear upregulation of p21 protein (see Fig. 2g and Supplemental Fig. S4). Western blot analysis, probing for the apoptotic marker cleaved caspase-3, shows our tumor and clone 5 cells, but not MEF cells, have increased apoptosis several days after 4-OHT treatment (see Fig. 2g and Supplemental Fig. S4). These data suggest that the loss of SMG7 may inhibit cell cycle progression and increase apoptosis in our RMS cells, but not in our MEF cells.

As our tumor and clonal cells exhibited an increased expression of p21, this may indicate a decrease in the fraction of actively proliferating cells. To analyze the effects of SMG7 loss on cell proliferation, we performed a 5-Chloro-2'-deoxyuridine (CldU) stain of our tumor and clonal cells 6 days after treatment. The percentage of RMS cells that had CldU^+^ nuclei per DAPI^+^ nuclei was significantly decreased in both our tumor (EtOH 57.44% ± 3.483 & 4-OHT 36.31% ± 0.9095; *P* = 0.0011) and clone 5 (EtOH 49.68% ± 2.374 & 4-OHT 36.13% ± 1.126; *P* = 0.0060) cells (see Fig. 2i,j and Supplemental Fig. S4). These data indicate that the loss of SMG7 induces a proliferation defect in our RMS cells.

As the RMS cells also exhibited cleaved caspase-3 staining, we further examined changes in cell death and apoptosis by utilizing propidium iodide (PI) and terminal deoxynucleotidyl transferase dUTP nick end labeling (TUNEL) assays. Our PI assay of day 6 treated cells showed a significant increase in the sub-G_0_/G_1_ peak in both the tumor (EtOH 2.12% ± 0.281 & 4-OHT 9.36% ± 1.63; *P* = 0.0120) and clone 5 (EtOH 3.54% ± 0.274 & 4-OHT 22.2% ± 5.46; *P* = 0.0142) cells (see Fig. 2k,l and Supplemental Fig. S4). Our TUNEL assay further confirmed an increase of apoptosis on day 6. The percentage of RMS cells that had TUNEL^+^ nuclei per DAPI^+^ nuclei was significantly increased in both the tumor (EtOH 0.685% ± 0.124 & 4-OHT 6.31% ± 1.61; *P* = 0.0318) and clone 5 (EtOH 0.812% ± 0.203 & 4-OHT 7.17% ± 1.10; *P* = 0.0013) cells (see Fig. 2m,n and Supplemental Fig. S4). These data suggest our RMS cells require SMG7 for full proliferative capacity and that the loss of SMG7 results in inhibited cell proliferation and increased apoptosis.

### SMG7 is required for full NMD activity

**Figure 3:**
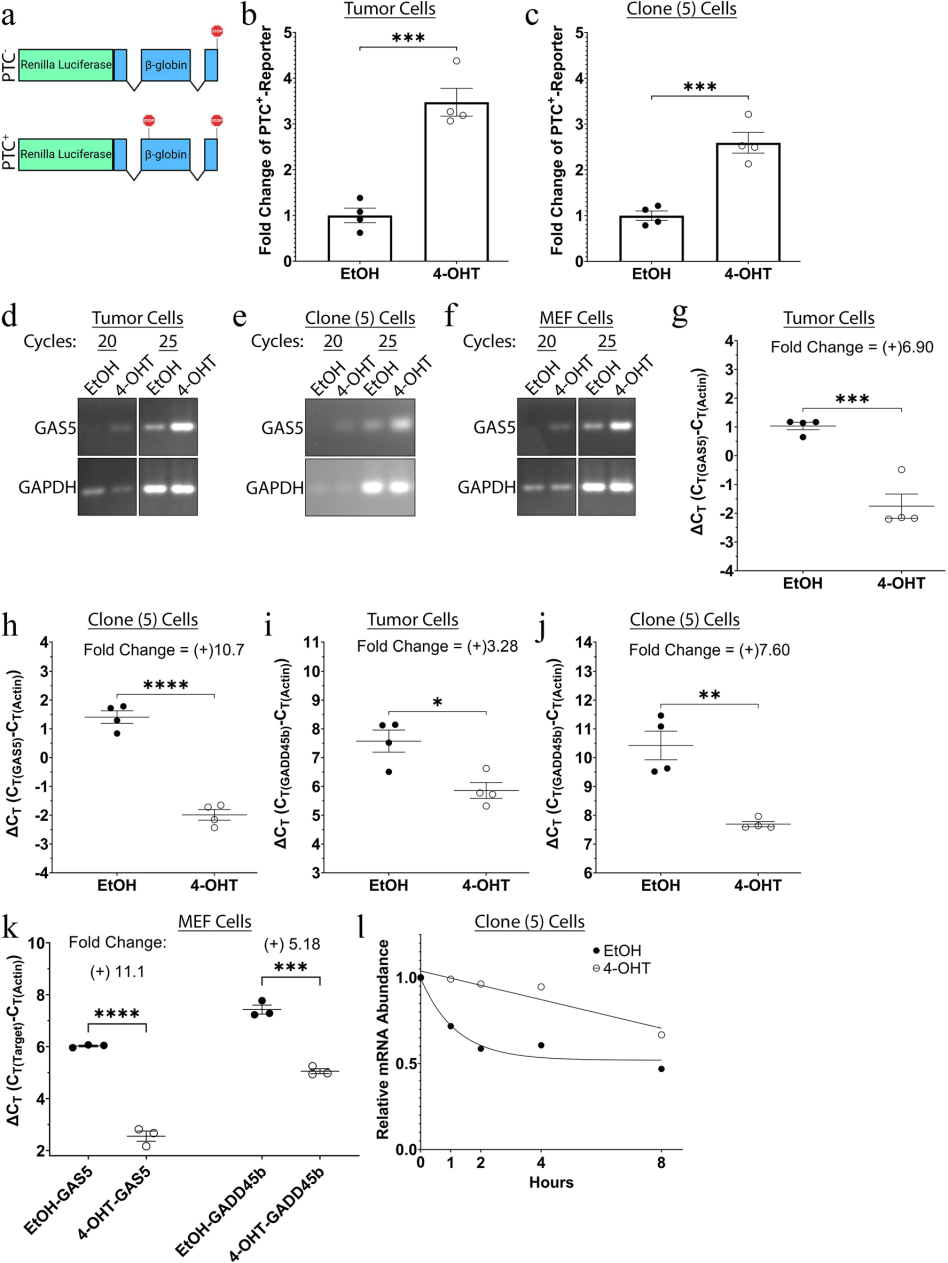
Loss of SMG7 inhibits NMD in RMS cells. (**a**–**c**) A schematic depicting the PTC^–^- (top) and PTC^+^- (bottom) luciferase constructs that were utilized to test changes in NMD activity is shown in (**a**). Tumor (**b**) and clone 5 (**c**) cells were transfected with luciferase plasmids 6 days after EtOH/4-OHT treatment. The relative luciferase activity of the constructs was measured on day 7 and normalized to the firefly luciferase. Quantifications in (**b** and **c**) show fold changes in the PTC^+^-reporter relative to EtOH treated cells from multiple, independent experiments. (**d**–**f**) Semi-quantitative PCR of day 6 EtOH/4-OHT treated tumor (**d**), clone 5 (**e**), and MEF (**f**) cells show amplified GAS5 and GAPDH bands after 20 and 25 cycles. The gels in (**d**) and (**f**) are cropped blots from the same gel. Cropped gels are shown in (**d**–**f**) and the original gels are available at the end of the Supplemental Figures. (**g** and **h**) GAS5 qPCR analysis of day 6 EtOH/4-OHT treated tumor (**g**) and clone 5 (**h**) cells are reported as ΔCt with the relative fold changes marked above the statistics. Data shows multiple, independent experiments. (**i** and **j**) GADD45b qPCR analysis of day 6 EtOH/4-OHT treated tumor (**i**) and clone 5 (**j**) cells are reported as ΔCt with the relative fold changes marked above the statistics. Data shows multiple, independent experiments. (**k**) GAS5 and GADD45b qPCR analysis of day 6 EtOH/4-OHT treated MEF cells are reported as ΔCt with the relative fold changes marked above the statistics. Data shows multiple, independent experiments. (**l**) Day 6 EtOH/4-OHT treated clone 5 cells were treated with 10 μM actinomycin D and RNA was collected at the indicated time points (0, 1, 2, 4, and 8 h). GAS5 qPCR analysis was performed and the mRNA abundance of multiple, independent experiments was plotted and fitted with a non-linear regression curve, which is shown in (**l**). (Unpaired 2-tailed t-tests were used for [**b** and **c** and **g**–**k**] with means ± SEM shown; A non-linear regression analysis was utilized in (**l**); N ≥ 3; *P < 0.05; **P < 0.01; ***P < 0.001; ****P < 0.0001; ns = *P* > 0.05).

Although SMG7 is known for its integral role in NMD, there are conflicting reports on the absolute necessity of SMG7 for full NMD activity^17,71,72^. It is unclear if other NMD machinery will be able to compensate for the loss of SMG7 in our cells. Therefore, we set out to confirm that the loss of SMG7 significantly decreases NMD activity in our RMS cells. To do this, we utilized a frequently used chemiluminescence-based NMD reporter assay that exploits PTC^+^- and PTC^–^-luciferases (see Fig. 3a)^73^. Analysis of the PTC^+^-luciferase on day 7 showed that both the tumor (EtOH 1.000% ± 0.1590 & 4-OHT 3.475% ± 0.3038; *P* = 0.0022) and clone 5 (EtOH 1.000% ± 0.1023 & 4-OHT 2.591% ± 0.2262; *P* = 0.0463) cells presented a ~ threefold significant increase in the PTC^+^-containing luciferase, indicating that SMG7 is required for full NMD activity in our RMS cells (see Fig. 3b,c). To further explore the role of SMG7-mediated NMD in our cells, we analyzed the expression of two specific NMD targets, GADD45b and GAS5. Both GADD45b and the lncRNA GAS5 are NMD targets that are associated with growth arrest and apoptosis^21–23^. We performed semi-quantitative PCR and real-time quantitative PCR (qPCR) on day 6 of our treated cells to see if the loss of SMG7 inhibited the NMD-mediated regulation of these two targets. Semi-quantitative PCR of our tumor, clone 5, and MEF cells showed a clear upregulation of GAS5 expression after 4-OHT treatment (see Fig. 3d–f). Furthermore, qPCR of our cells showed a significant upregulation of GAS5 expression in our tumor (ΔCt—EtOH 1.033 ± 0.1285 & 4-OHT − 7.754 ± 0.4231; *P* = 0.0007), clone 5 (ΔCt—EtOH 1.409 ± 0.2187 & 4-OHT − 1.987 ± 0.1848; *P* = 0.00002), and MEF (ΔCt—EtOH 6.027 ± 0.0335 & 4-OHT 2.554 ± 0.1978; *P* = 0.00007) cells after the loss of SMG7 (see Fig. 3g,h,k). The qPCR analysis of our cells also showed a significant upregulation of GADD45b expression in our tumor (ΔCt—EtOH 7.576 ± 0.3837 & 4-OHT 5.863 ± 0.2744; *P* = 0.0110), clone 5 (ΔCt—EtOH 10.43 ± 0.4973 & 4-OHT 7.695 ± 0.0909; *P* = 0.0017), and MEF (ΔCt—EtOH 7.429 ± 0.1711 & 4-OHT 5.056 ± 0.0966; *P* = 0.0003) cells after the loss of SMG7 (see Fig. 3.i,j,k). To further confirm SMG7’s role in NMD-mediated regulation of GAS5, we analyzed the mRNA stability of GAS5 in our RMS cells. Six days after treatment, we inhibited transcription in our clone 5 cells through actinomycin D treatment and measured the mRNA abundance of GAS5 at several time points. We fit a non-linear regression curve to EtOH and 4-OHT treated cells to analyze the decay rates. The EtOH treated clone 5 cells exhibited a normal NMD-mediated biphasic decay of GAS5 (see Fig. 3l). The 4-OHT treated clone 5 cells switched from the fast, biphasic decay seen in the EtOH treated cells to a much slower linear decay (see Fig. 3l). These data suggest that SMG7 plays a crucial role in NMD in our RMS and MEF cells.

### SMG7 is crucial for full RMS tumor growth

**Figure 4:**
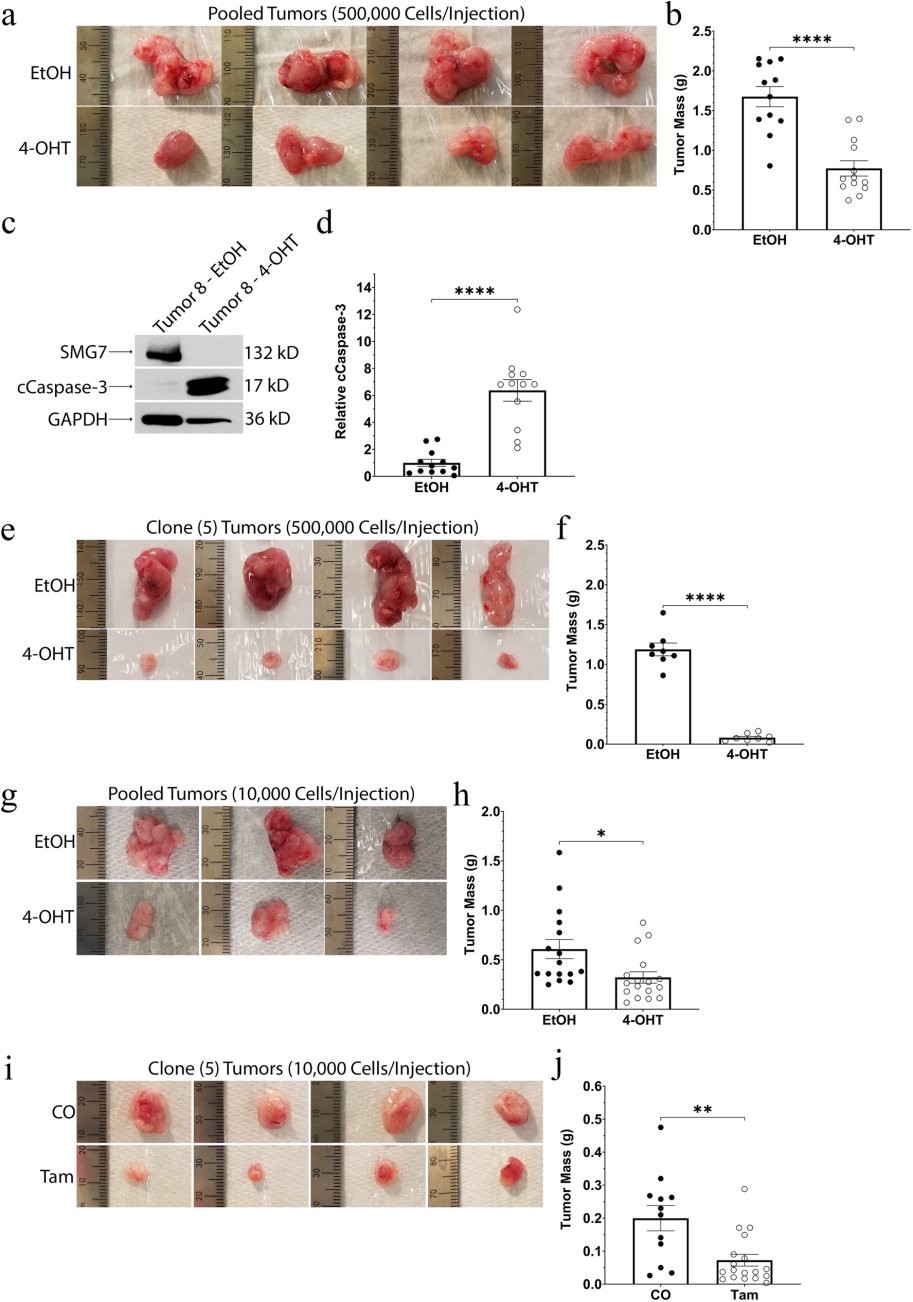
Loss of SMG7 inhibits tumor growth of RMS cells. (**a**–**d**) Tumor cells were treated with EtOH/4-OHT for 2 days before 500,000 cells were subcutaneously injected into the flanks of mice (EtOH treated cells injected into the right flank and 4-OHT treated cells injected into the left flank) and allowed to grow for 10 days before we sacrificed the mice and collected their tumors. Images of representative, paired tumors are shown in (**a**). The masses of collected tumors are quantified in (**b**). A western blot of representative tumors is shown in (**c**). The relative levels of cleaved caspase-3 are normalized to EtOH pretreated tumors and are quantified in (**d**). Cropped blots are shown and the original are available at the end of the Supplemental Figures. (**e** and **f**) Clone 5 cells were treated with EtOH/4-OHT for 2 days. The cells were passaged and allowed to grow for 3 more days before 500,000 cells were subcutaneously injected into the flanks of mice (EtOH treated cells injected into the right flank and 4-OHT treated cells injected into the left flank) and allowed to grow for 10 days before we sacrificed the mice and collected their tumors. Images of paired, representative tumors are shown in (**e**). The masses of collected tumors are quantified in (**f**). (**g** and **h**) Tumor cells were treated with EtOH/4-OHT for 2 days before 10,000 cells were subcutaneously injected into the flanks of mice (EtOH treated cells injected into the right flank and 4-OHT treated cells injected into the left flank) and allowed to grow for 14 days before we sacrificed the mice and collected their tumors. Images of representative, paired tumors are shown in (**g**). The masses of collected tumors are quantified in (**h**). (**i** and **j**) 10,000 clone 5 cells were subcutaneously injected into the flanks of mice and allowed to grow for 3 days. Then the mice received intraperitoneal injections of corn oil (CO) or Tamoxifen (Tam) on 3 consecutive days. Mice were sacrificed 14 days after the initial clone 5 injection and their tumors were collected. Images of representative tumors are shown in (**i**) and the mass of individual tumors are quantified in (**j**). (Unpaired 2-tailed t-tests with means ± SEM are provided in quantifications; (**a**–**d**) N = 12 mice; (**e** and **f**) N = 8 mice; (**g** and **h**) N = 17 mice; (**i** and **j**) N = 12 CO treated mice & N = 18 Tam treated mice; *P < 0.05; **P < 0.01; ****P < 0.0001).

As the loss of SMG7 significantly decreased the fitness of our RMS cells in vitro, we then turned to in vivo studies to test if the loss of SMG7 would have a similar inhibitory phenotype on RMS tumor growth. We have shown that our RMS cells have the capacity to form large tumors over a short period of time with 100% penetrance, regardless of low cell counts (see Fig. 1c,d,f,g). To analyze the importance of SMG7 for tumor growth, we performed several in vivo experiments. We pretreated the tumor cells for two days and then subcutaneously injected 500,000 tumor cells into the flanks of mice. Ten days after the injection, we sacrificed the mice and collected their tumors. The loss of SMG7 significantly decreased the tumor growth of our RMS cells, as exhibited by a ~ 54% lower average tumor mass in the 4-OHT treated group (EtOH 1.675 g ± 0.1264 & 4-OHT 0.7728 g ± 0.09637; *P* = 0.000007) (see Fig. 4a,b). Western blot analysis of the tumors showed that the 4-OHT pretreated group exhibited a ~ 6.4-fold significant increase in the levels of cleaved caspase-3 (EtOH 1.000 ± 0.2627 & 4-OHT 6.370 ± 0.8001; *P* = 0.000002) (see Fig. 4c,d), recapitulating our in vitro studies. In a similar experiment, we pretreated two of our clonal cell lines for two days, allowed them to expand for three more days, and then subcutaneously injected the cells into the flanks of our mice. The tumors were allowed to develop for 10 days before we sacrificed the mice and collected their tumors. The loss of SMG7 significantly decreased tumor growth in both of the clonal populations, as evidenced by their average tumor masses. The 4-OHT pretreated cells showed a ~ 49% decrease in the average mass of clone 1 tumors (EtOH 1.132 g ± 0.1237 & 4-OHT 0.5809 g ± 0.1098; *P* = 0.0060) and a ~ 93% decrease in the average mass of clone 5 tumors (EtOH 1.188 g ± 0.0798 & 4-OHT 0.0822 g ± 0.0172; *P* = 0.000000002) (see Fig. 4e,f and Supplemental Fig. S5). To further analyze and categorize the effects that the loss of SMG7 has on tumor growth in our RMS cells, we repeated the 2-day pretreatment-experiment using a smaller fraction of tumor and clonal cells. We pretreated our RMS cells for 2 days and subcutaneously injected 10,000 tumor or clone 5 cells into the flanks of mice. The tumors were allowed to develop for 14 days, at which point we sacrificed the mice and collected their tumors. Although we used a smaller number of RMS cells, the EtOH pretreated groups developed large tumors and the 4-OHT pretreated group exhibited a significantly smaller average tumor mass, in both the tumor cell derived tumors (EtOH 0.6079 g ± 0.0966 & 4-OHT 0.3214 g ± 0.0577; *P* = 0.0148) and the clone 5 cell derived tumors (EtOH 0.3276 g ± 0.0399 & 4-OHT 0.1203 g ± 0.0188; *P* = 0.00005) (see Fig. 4g,h and Supplemental Fig. S5)*.* Deletion of SMG7, induced by 4-OHT treatment, significantly decreased tumor growth of our RMS cells, regardless of the number of tumor cells we initially injected. These data suggest that SMG7 is a major player in tumor growth and the cellular fitness of our RMS cells, both in vitro and in vivo.

As our pretreatment experiments showed that SMG7 plays an important role in the tumor growth of our sarcoma cells, we wanted to explore the viability of targeting SMG7 in vivo. Clinical studies have shown that the treatment of RMS has benefited from a neoadjuvant therapy approach, because of this, we applied a similar method to our in vivo studies^74–76^. To do this, we subcutaneously injected 10,000 clone 5 cells into the flanks of our mice. As we have shown that our RMS cells have a robust tumor-initiating capacity, we allowed the cells to grow for three days before we initiated corn oil (CO) or tamoxifen (Tam) treatment on three consecutive days. Fourteen days after the initial injection of RMS cells, we sacrificed the mice and collected their tumors. The Tam treated group had an average tumor mass that was ~ 63% lower than the average tumor mass of the CO treated group (CO 0.1998 g ± 0.0382 & Tam 0.0725 g ± 0.0178; *P* = 0.0023) (see Fig. 4i,j), giving credence to the possibility of targeting SMG7 function for STS treatment. These tumor studies show that SMG7 is required for the full capacity tumor growth of our RMS cells and, without SMG7, tumors exhibit increased levels of apoptosis and significantly inhibited tumor growth.

### SMG7 is dispensable in adult mice

While the effects of SMG7 knockout on the developing tumors were significant and salutary, we wanted to confirm that SMG7 knockout was not completely deleterious to adult mice. To do this, we injected adult mice with CO or Tam on three consecutive days. Fourteen days after the initial injection, every mouse was alive and appeared healthy, at which point we sacrificed them and collected their organs. Analysis of the CO treated group showed that SMG7 is expressed at high levels in brain and testicular tissues, at medium levels in lung, heart, and skeletal muscle tissues, and at low levels in spleen tissue (see Supplemental Fig. S6). SMG7 expression was undetectable in the tissues of the liver, stomach, kidney, and small and large intestines (see Supplemental Fig. S6). The Tam treated mice exhibited a significantly smaller ratio of thymus mass/body mass (CO 0.1047% ± 0.0149 & Tam 0.0429% ± 0.0064; *P* = 0.0044) (see Supplemental Fig. S6). The Tam treated mice had a slight but significant increase in the ratio of lung mass/body mass (CO 0.6201% ± 0.0138 & Tam 0.6980% ± 0.0202; *P* = 0.02) (see Supplemental Fig. S6). The Tam injected mice also had a large and significant increase in the ratio of stomach mass/body mass (CO 0.9042% ± 0.0767 & Tam 2.417% ± 0.125; *P* = 0.00003) (see Supplemental Fig. S6). Interestingly, the Tam treated mice did not have a significant change in the ratio of testicle mass to body mass (CO 0.5191 ± 0.0508 & Tam 0.5237 ± 0.0216; *P* = 0.9309) (see Supplemental Fig. S6). This is in contrast to the drastic effect UPF2 knockout exhibited on testicle development in postnatal, juvenile mice^70^. Other examined organs included the brain, heart, liver, spleen, kidney, and quadriceps femoris muscle, none of which exhibited a significant change in the ratio of organ mass/body mass after Tam treatment (see Supplemental Fig. S6). While our knockout of SMG7 in adult mice was a short-term study, the mice appeared healthy at the time of sacrifice. These data suggest that SMG7 is dispensable in adult mice and has few effects on the gross anatomy of mouse organs.

## Discussion

In this study, we established and characterized a STS primary cell line derived from our GEMM. We identified our STS cell line as a pleomorphic RMS. The loss of SMG7 significantly upregulated NMD target genes in both our RMS and MEF cells. Notably, the loss of SMG7 significantly inhibited proliferation and induced apoptosis in our RMS cells but not in our MEF cells. Our mouse studies showed that deletion of SMG7 significantly decreased tumor growth of our RMS cells and that SMG7 was dispensable for adult mouse survival, which is in contrast to UPF2, where the conditional knockout of UPF2 in adult mice quickly led to death^67^. These data suggest that SMG7 may act as a cancer fitness gene in RMS and may be a feasible target for cancer therapy.

GEMMs, along with PDXs, are considered to be the most representative and therapeutically predictive models for studying STSs^52^. Our SMG7 conditional knockout GEMM lacks p53, and in conformity with this genotype, one of our mice spontaneously developed a pleomorphic RMS. Pleomorphic RMSs mostly affect older patients, have a poor prognosis, a high rate of recurrence, and are associated with p53 mutations both in humans and in mice^35,77–82^. Although our data reflect a sanguine discovery for SMG7 as a novel therapeutic target for RMS treatment, it is important to note the limitations of our study. Our study reflects the crucial role that SMG7 plays in one RMS tumor population. As STSs are heterogeneous both as a whole and within subtypes, extrapolating our results across the RMS and STS fields would be inappropriate. Further analysis of SMG7 as a therapeutic target in RMS would benefit from PDX studies, which better reflect the heterogeneity and unique response to treatments that arise in individual patients.

It is unclear exactly why SMG7 is required for RMS proliferation and survival but is dispensable for MEF viability and adult mouse homeostasis. The NMD pathway is complex, and we are nescient regarding its complete involvement in development, differentiation, and homeostasis. SMG7 is known to be required for embryogenesis in *A. thaliana* and *D. rerio*^54,55^. Similarly, several other NMD factors are required for embryogenesis in *Mus musculus*^53,56–59^. In *M. musculus* and in *Xenopus laevis*, the loss of NMD factors induced embryogenesis defects that presented as early as blastocyst implantation, continued throughout gastrulation, and into early organogenesis^53,56,57,59,68^. These studies suggest that NMD factors are dispensable during totipotency but are essential for differentiating out of pluripotency. In support of this, knockout of SMG5, SMG6, or SMG7 in naïve mouse embryonic stem cells showed that these NMD factors were dispensable for in vitro proliferation; however, the loss of any one of these NMD factors produced defects in exiting from both naïve and general pluripotency^83^. Similarly, pre-implantation mouse blastocysts null for UPF1 were able to proliferate unabated in vitro for 48 h^57^. By 72 h, however, the inner cell mass showed heavy induction of apoptosis^57^. Likewise, in mouse embryonal carcinoma and human embryonic stem cells, the loss of UPF1 significantly decreased stem cell markers and inhibited proliferation^68,84^. Finally, in human embryonic stem cells, NMD appears to be downregulated for endodermal differentiation and upregulated for ectodermal/mesodermal differentiation^84^. Additionally, a broad RNA-seq analysis of human cell lines showed that some NMD factors are significantly downregulated in non-pluripotent cells compared to pluripotent cells, further suggesting a divergent role for NMD in differentiation and homeostasis^84^. Based on these studies, we speculate that tightly-regulated NMD may be required to navigate pluripotency but be dispensable for more differentiated cells, including multipotent cells. If so, this may explain the phenotype whereby our MEF cells and adult mice do not require SMG7 for viability and homeostasis, respectively. Supporting our speculation, RMS patient samples express several pluripotent cancer stem cell markers (CD24, CD133, Oct-4, Sox2, Nanog, and c-Myc) and are able to differentiate into neuronal cells, osteogenic cells, myocytes, and adipocytes^85–87^. In contrast, MEF cells exhibit a more differentiated multipotent stem-like state, characterized by a lack of pluripotent stem cell markers (CD133, Oct-4, Sox2, and c-Myc)^64–66^. Similarly to our MEF cells, the loss of SMG7 in our adult mice had no effect on mouse survival, which is in contrast to the macabre results seen when knocking out UPF2 in the hematopoietic population of adult mice, suggesting that SMG7 is dispensable for the multipotent hematopoietic population^67^. Accordingly, the loss of SMG7 may be deleterious to the pluripotent RMS cells but be dispensable for the multipotent MEF and adult mouse stem cells.

SMG7 may play a pro-tumorigenic role in our RMS cells by downregulating stress inducing PTC^+^-transcripts. NMD acts as a quality control and transcriptomic regulatory mechanism by clearing PTC^+^-transcripts from the cell. Importantly, knocking down UPF1 in CRC cells with microsatellite instability (MSI) induced the expression of hundreds of mutant PTC^+^-transcripts with concomitant inhibition of proliferation and tumor growth, while the growth of CRC cells without MSI was unaffected^44^. This phenotype may also explain the differential response to the loss of SMG7 that we observed in our RMS and MEF cells. Upregulation and translation of mutant or endogenous PTC^+^-transcripts in our RMS cells may create a plethora of proteotoxic misfolded proteins that induce ER stress that, in turn, activates the integrated stress response (ISR) and the unfolded protein response (UPR) pathways. Activation of these pathways may further inhibit residual NMD activity through a positive feedback loop^88–90^. The additional loss of NMD, combined with failure of the ISR and UPR pathways to turn off, may induce cell cycle arrest and apoptosis in our RMS cells^38,88,91–93^. The lack of deleterious mutations, coupled with divergent cell-type-specific signalling and responses, in our MEF cells may mean that the quality control and transcriptomic regulatory mechanisms of NMD are not required to maintain cell viability. Overall, if this speculation is accurate and similar to what was seen in MSI CRC cells, then a cautionary note is appropriate regarding the applicability of our findings to STSs commonly seen in the clinic. While a subset of RMSs have been described as displaying a high tumor mutation burden, there is ordinarily a low tumor mutation burden and low incidence of MSI seen in pleomorphic RMSs, RMSs, and STSs^9,27,82,94–96^.

SMG7 may play a pro-tumorigenic role in our cells by downregulating several anti-cancer genes. We have observed that SMG7 is required for targeting GADD45b and the tumor suppressor lncRNA GAS5 for degradation in our cells (see Fig. 3d–l). Upregulation of these anti-cancer genes was concomitant with inhibition of proliferation and induction of apoptosis in our RMS cells. Notably, upregulation of these anti-cancer genes had no effect on our primary MEF cells. In a previous report, UPF1 knockdown in NIH-3T3 cells induced upregulation of GADD45b with a concomitant induction of apoptosis, which was almost completely rescued after double knockdown of UPF1 and GADD45b^23^. The differential effects seen in the NIH-3T3, RMS, and primary MEF cells may be the result of several combinatorial factors that are cell-line/cell-type specific. Firstly, our MEF cells were examined at a relatively low passage number and were unlikely to contain many mutations or chromosomal aberrations. In contrast, both the NIH-3T3 and our RMS cells have acquired mutations and/or chromosomal abnormalities to become immortalized or tumorigenic, respectively. These genomic alterations may add additional, unaccounted for stresses that cause the loss of SMG7 to become lethal. Secondly, tissue-specific gene expression and signalling most likely influence our observed differential phenotype. Supporting this, the loss of SMG7 in our adult mice produced disparate effects across organs. While our Tam treated mice exhibited no observable changes in the gross anatomy of most of their organs, they did display significant changes in the ratio of organ mass over body mass of thymi, lungs, and stomachs, in comparison to EtOH treated mice (see Supplemental Fig. S6). Atrophy of the thymus due to reduced NMD is not surprising given the T-cell-specific requirement for NMD to suppress deleterious PTC^+^-TCR-β transcripts that may arise during V(D)J recombination^97^. Similar to our observed phenotype, disruption of NMD during mouse fetal development induced intense levels of apoptosis and was concomitant with disrupted thymic architecture^97^. Importantly, observed organ abnormalities were limited to the thymus, suggesting a tissue-specific sensitivity or requirement for NMD^97^. Our Tam treated mice also had a small but significant increase in the ratio of lung mass/body mass (see Supplemental Fig. S6). Our data show a moderate amount of SMG7 expression in the lungs (see Supplemental Fig. S6), suggesting that the loss of SMG7 may have led to this increase in lung mass. In contrast to this speculation, a previous study has shown that expression of NMD factors in the lungs is rather low, with a concomitant low efficiency of NMD^98^. Although our Tam treated mice exhibited a large and significant increase in the ratio of stomach mass over body mass (see Supplemental Fig. S6), this is most likely due to the stimulatory effects that Tam has on stomach cell proliferation, rather than the loss of SMG7^99–101^. This claim is further corroborated by our mice having no detectable expression of SMG7 in their stomachs (see Supplemental Fig. S6), as well as by a previously described low expression of NMD factors and NMD efficiency in the stomachs of mice^98^. Interestingly, the Tam treated mice did not produce a significant change in the ratio of testicle mass to body mass (see Supplemental Fig. S6). This is in contrast to the drastic effect UPF2 knockout exhibited on testicle development in juvenile mice, further suggesting that NMD is required for specific cell-types during development but may be dispensable for adult homeostasis^70^. Accordingly, divergent cell-type-specific gene expression and signalling may account for how the loss of SMG7 and upregulation of GADD45b/GAS5 may induce differential effects in our RMS cells, MEF cells, and adult mice.

In this study, we have shown that SMG7 behaves similarly to a cancer fitness gene. Though a “cancer fitness gene” is a relatively novel concept, the defining features are (I) they do not initiate transformation like driver oncogenes, (II) they enhance tumor progression through proliferative, survival, stress-response, or metastatic advantages, and (III) they are generally less essential for normal tissues under homeostatic conditions^10^. Our data supports SMG7 as a cancer fitness gene under (II) and (III), as we showed SMG7 was critical to maintain RMS growth and survival (II) but was dispensable for MEFs and adult mice (III); however, the role of SMG7 in transformation (I) is currently unknown. While there is evidence to support the idea that NMD factors act in either a pro- or anti-tumorigenic role in divergent cancers, the necessity for SMG7 in transformation is unexplored^11,12,26,29,37–48,102,103^. In total, current evidence suggests that SMG7 is a part of a complex pathway that may be a tool cancer cells utilize for a proliferative, survival, stress-response, or metastatic advantage.

Future studies aim at parsing out the mechanism that induces inhibited proliferation and increased apoptosis in our RMS cells following the loss of SMG7. We hypothesize the anti-tumor effects seen following SMG7 knockout in our RMS cells result from the loss of NMD and its mediated suppression of PTC^+^-transcripts, cell cycle arrest genes, and pro-apoptotic genes. Of prime interest are GADD45b and the tumor suppressor GAS5. Other future studies aim at utilizing our novel GEMM to explore the role of SMG7 in other subtypes of STS as well as what role, if any, SMG7 plays in initial sarcomagenesis.

In conclusion, we present here that SMG7 plays a critical role in RMS proliferation and survival but is dispensable for MEF viability and adult mouse homeostasis. Our data evinces SMG7 as a prospective cancer fitness gene in RMS and that disrupting SMG7 function may be tolerable and provide a therapeutic benefit for patients with STS.

## Methods

### Development of the genetically engineered mouse model (GEMM)

We developed a SMG7 conditional knockout mouse (C57BL/6 background) using the knockout-first design (see Supplemental Fig. 1)^60,61^. In short, the second exon of *Smg7* is flanked by loxP sites, resulting in tm1c mice. We crossed the *Smg7*^*tm1c*^*/Smg7*^*tm1c*^ mice with *ROSA26*^*CreER*^ mice to achieve inducible-knockout *Smg7*^*tm1c*^*/ Smg7*^*tm1c*^*; ROSA26*^*CreER*^*/ROSA26*^*CreER*^ mice (*Smg7*^*flox/flox*^). Induction of Cre-mediated recombination results in *Smg7*^*tm1d*^*/Smg7*^*tm1d*^ alleles (*Smg7*^*Δ/Δ*^), which encodes for a truncated SMG7 product that is undetectable by western blot. The *Smg7*^*flox/flox*^ mice were then crossed with *Trp53*^*−/−*^ (also known as *p53*^*−/−*^) (C57BL/6 background) mice until *Smg7*^*flox/flox*^*; p53*^*−/−*^ mice were acquired.

### Isolation of primary soft-tissue sarcoma cells

Tumor bearing mice were euthanized using a CO_2_ chamber. CO_2_ flow was maintained for at least 60 s after respiratory arrest and was followed by physical death verification by bilateral thoracotomy. After euthanasia, mice were sterilized with 70% ethanol (111,000,200), and transferred to a biosafety hood. Tumors were excised and washed in 1× PBS (20–031-CV). Excised tumors were minced in a slurry of collagenase/dispase (10,269,638,001) and trypsin (0.5%) (25–053-CI). Tumor solution was incubated at 37 °C for 30 min to release tumor cells. 5 mL DMEM (10–013-CV) (10% FBS [F2442] + 1% penicillin–streptomycin [30–002-CI]) was added to neutralize proteases. Tumor cells were filtered through a 70 μm filter (352,350) and centrifuged at 50 × g for 5 min. The supernatant was aspirated, the cell pellet was suspended in DMEM (10% FBS + 1% penicillin–streptomycin), and the cells were manually counted using a hemocytometer. 5 × 10^6^ tumor cells were plated in 10 cm plates (229,621). After the tumor cells were isolated, aliquots of the pooled tumor cell population were frozen. To generate clones, the pooled tumor cell population was split into 96-well plates by limiting dilution (final concentration − 100 cells/96-well plate). Colonies were allowed to develop over a period of 2 weeks while we constantly monitored for and marked individual/multiple clone colonies. Individual clone colonies were expanded to 12-well plates, then 6 cm, and finally 10 cm plates. Pooled and clonal tumor cells were maintained for five passages before freezing and validating. To confirm the cells were of skeletal muscle lineage, the pooled and clonal tumor cells were stained with MyoD1. To validate that the tumor cells retained their tumor-initiating capacity, the pooled and clonal tumor cells were subcutaneously injected into the flanks of mice (4 mice per cell line). Tumors were collected, fixed, and then stained with H&E. The histological subtypes of tumors were characterized based on the WHO Classification of Tumours (*Soft Tissue and Bone Tumours*)^5^.

### Isolation of MEF cells

Pregnant mice were sacrificed on embryonic day 13.5 using a CO_2_ chamber. CO_2_ flow was maintained for at least 60 s after respiratory arrest and was followed by physical death verification by bilateral thoracotomy. After euthanasia, mice were sprayed with 70% ethanol to sterilize them, and then transferred to a biosafety hood. Uteri were isolated and embryos individualized. The heart, hands, feet, and tail were removed from the embryo. Then the embryo was diced until it was able to be pipetted. Trypsin (0.25%) was mixed with the chopped embryo for 15 min. After incubation, cells were collected, resuspended in fresh media, and plated. The next day, fresh, warmed media was given to MEF cells.

### Cell culture

Primary rhabdomyosarcoma (RMS) and mouse embryonic fibroblast (MEF) cells were maintained in DMEM (10% FBS). RMS and MEF cells were maintained at sub-confluent levels and passaged every 2–3 days.

### Cre-induced knockout of SMG7

RMS and MEF cells were used for treatment only if they were low passage (< 10 passages). RMS and MEF cells were treated with 4-hydroxytamoxifen (2.58 μM) (H6278) or ethanol (3.43 mM) dissolved in DMEM (10% FBS) for 48 h. After 48 h, cells were washed with 1× PBS and passaged for downstream experiments.

For in vivo knockout of SMG7, age matched mice (2- to 3-month-old) received intraperitoneal injections (100 μL) of tamoxifen (27 mM) or ethanol (0.857 M) corn oil on three consecutive days. Treatment of all mice occurred at midday and in their home cage. At the endpoint of experiments, mice were euthanized using a CO_2_ chamber. CO_2_ flow was maintained for at least 60 s after respiratory arrest and was followed by physical death verification by bilateral thoracotomy.

### Tumor-initiating capability of primary tumor cells

Primary tumor or clonal cells were trypsinized, collected, washed with PBS (2×), counted using a hemocytometer, and resuspended at the appropriate concentration for a final injection volume of 50 μL (× cells/50 μL). Age matched male mice (2- to 3-month-old) were used for all experiments. Injection of all mice occurred at midday and in their home cage. Mice were wiped down with 70% ethanol saturated gauze to sterilize the injection site before receiving a subcutaneous injection with 50 μL of cell slurry (500,000 or 10,000 cells total). At the endpoint of experiments, mice were euthanized using a CO_2_ chamber. CO_2_ flow was maintained for at least 60 s after respiratory arrest and was followed by physical death verification by bilateral thoracotomy. The mass, length and width of tumors were measured before tumor tissues were processed for downstream analysis.

### MyoD stain

Primary tumor cells were plated on poly-l-lysine coated coverslips and allowed to adhere overnight. The next day, the media was aspirated, the cells were washed with PBS (2×) and then fixed with ice-cold methanol for 10 min. After fixation, methanol was aspirated, samples were rinsed with PBS (2×) and then blocked with 2% BSA (BP1600-100) 0.1% tween-20 (P1379) PBS (PBST) for 30 min. After blocking, samples were rinsed with PBS (2×) and then incubated with anti-MyoD primary antibody (Mouse—sc-377460) overnight. The next day, the primary antibody solution was reclaimed, samples were rinsed with PBS (2×) and then washed with 2% BSA PBST for 30 min. After washing, PBST was aspirated, cells were washed with PBS (2×) and then incubated with Alexa Fluor 488 anti-Mouse secondary antibody (Goat—A-11001) for 30 min. After incubation, secondary antibody was reclaimed, samples were rinsed with PBS (2×), and then washed with 2% BSA PBST for 30 min. After washing, samples were rinsed with distilled water (2×) before counterstaining with DAPI (23 nM) (10,236,276,001) for 10 min at room temperature. After incubation, DAPI solution was aspirated and samples were rinsed with water (2×) before coverslips were mounted to slides with ProLong™ Diamond Antifade Mountant (P36970). Slides were imaged on an Olympus BX61 upright fluorescent microscope and the images were analyzed using ImageJ software.

### Crystal violet stain

RMS and MEF cells were treated with 4-hydroxytamoxifen (4-OHT) or ethanol (EtOH) as above. Forty-eight hours after treatment, tumor cells were passaged and plated on 24-well plates (229,123). Cells were stained 3 and 6 days after initial treatment. For staining, culture media was aspirated and cells were washed with 1× PBS. The PBS was aspirated and cells were incubated with 1 mL methanol (A411-4) for 10 min. Methanol was aspirated and 300 µL of crystal violet (C0775) solution was added to cells for 20 min. Crystal violet solution was aspirated and cells were washed with distilled water 4 times. The plate was allowed to dry overnight. For optical density quantification, 1 mL of methanol was added to each well and incubated for 20 min. The optical density was measured at 570 nm using a BioTek® Synergy 2 Multimode Plate Reader.

### Western blot

Protein was isolated using BC100 buffer (20 mM Tris–HCl [pH 7.3], 100 mM NaCl, and 10% glycerol with 1% SDS) and sonication. Protein concentrations were estimated using a BSA curve and the Bio-Rad Protein Assay Dye Reagent (#5,000,006). Samples were resolved on a SDS-PAGE gel and transferred onto nitrocellulose membrane. Nitrocellulose membranes were stained with Ponceau S and cut at the appropriate molecular markers before blocking and primary antibody hybridization. The following antibodies were used for immunoblots: SMG7 (Rabbit—A302-170A), β-Actin (Mouse—sc-47778), GAPDH (Rabbit—D16H11), p21 (Mouse—sc-53870), cleaved Caspase-3 (Rabbit—#9661), anti-Rabbit HRP (Goat—7074S), and anti-mouse HRP (Sheep—NA931V). ImageJ software was used to quantify immunoblots. Photoshop was used for western blot image processing (Image→Adjustments→Black & White→Blue Filter→minimal contrast adjustments). Original, unaltered western blot images are available at the end of the Supplemental figures (see online).

### PCR, semi-quantitative PCR, and real-time quantitative PCR (qPCR)

PCR was performed on mouse tissues on a Veriti™ 96-Well Fast Thermal Cycler (4,375,305) with the following PCR conditions: 60 s at 95 °C followed by 36 cycles of 95 °C for 30 s, (Cre primer—60 °C & tm1c primers—58 °C) for 30 s, and 68 °C for 1 min and 15 s. Total RNA was extracted from cells using TRIzol™ Reagent (15,596,018) according to the manufacturer’s protocol. Reverse transcription was performed using the standard NEB protocol (#M0253 with M0253L). Semi-quantitative PCR was performed with Phusion Hot Start II DNA Polymerase (F-549L) on a Veriti™ 96-Well Fast Thermal Cycler (4,375,305) with the following PCR conditions: 30 s at 98 °C followed by 20, 25, and 30 cycles of 98 °C for 15 s, (Gapdh primers—60.6 °C & Gas5 primers—62 °C) for 15 s, and 72 °C for 1 min. The qPCR samples were run in triplicate utilizing PowerSYBR™ Green PCR Master Mix (4,367,659) on the StepOnePlus Real-Time PCR System (4,376,600) with the following PCR conditions: 10 min at 95 °C followed by 40 cycles of 95 °C for 15 s and (Gapdh/Actb/p21/Gadd45b primers—60.6 °C & Gas5 primers—62 °C) for 1 min. The following primers were used for PCR: Actb (5′-CCCTATAAAACCCAGCGGCGCGA-3′ and 5′-TCTCGCGGTTGGCCTTGGGG-3′), Gapdh (5′-AGGTCGGTGTGAACGGATTTG-3′ and 5′-TGTAGACCATGTAGTTGAGGTCA-3′), Cdkn1a (also known as p21) (5′-GTGGCCTTGTCGCTGTCTT-3′ and 5′-GCGCTTGGAGTGATAGAAATCTG-3′), Gas5 (5′-TTTCCGGTCCTTCATTCTGA-3′ and 5′-TCTTCTATTTGAGCCTCCATCCA-3′), and Gadd45b (5′-CTGATGAATGTGGACCCC-3′ and 5′-CCGGACGATGTCAATGTC-3′).

### NMD luciferase reporter assay

Constructs were obtained from Andreas Kulozik as a generous gift (pCL-Neo β-globin WT Renilla, pCL- Neo β-globin NT39 Mutant Renilla, and pCL- Neo Firefly)^73^. After initial treatment and passaging (see above), RMS cells were transfected on day 6 using Lipofectamine 2000 (11668-019). Half the samples received the *Renilla β-globin WT* and *Firefly* constructs and the other half of samples received the *Renilla β-globin NT39 Mutant* and *Firefly* constructs. The cells were harvested 1.5 days later and the relative luciferase activity was detected using the Dual Luciferase® Reporter Assay System (E1910) and a 20/20n Luminometer (2030-000). *Renilla* signals were normalized to *firefly* controls and the results were reported as fold change relative to ethanol treated cells.

### mRNA stability assay

A bio-protocol was used for the mRNA stability assays^104^. In short, RMS cells were treated with 10 uM Actinomycin D on day 6 and total RNA was collected at several time points (0, 1, 2, 4, and 8 h). After total RNA isolation and reverse transcription, qPCR was performed using Gapdh and Gas5 primers (see above), and mRNA abundances were calculated for each time point using multiple independent experiments for all time points. The relative mRNA abundances of each time point were plotted using GraphPad Prism 9 and the decay rate was determined by non-linear regression curve fitting (one phase decay).

### CldU stain

After initial treatment and passaging (see above), RMS cells were plated on poly-L-lysine coated coverslips on day 5 and allowed to adhere overnight. The next day, cells were fed 25 µM CldU (105,478) dissolved in warmed DMEM (10% FBS) and incubated for 20 min at 37 °C. After incubation, the media was aspirated, the cells were washed with PBS (2×) and then fixed with ice-cold methanol for 10 min. After fixation, methanol was aspirated, samples were rinsed with PBS (2×), and then incubated with 3 M HCl (A481-212) for 30 min at room temperature. After incubation, HCl was aspirated, samples were washed with PBS (2×), and then blocked with 2% BSA (BP1600-100) 0.1% tween-20 (P1379) PBS (PBST) for 30 min. After blocking, samples were rinsed with PBS (2×) and then incubated with anti-BrdU primary antibody (Rat—MCA2060) overnight. The next day, primary antibody solution was reclaimed, samples were rinsed with PBS (2×) and then washed with 2% BSA PBST for 30 min. After washing, PBST was aspirated, cells were washed with PBS (2×) and then incubated with Alexa Fluor 488 anti-Rat secondary antibody (Goat—A-11006) for 30 min. After incubation, secondary antibody was reclaimed, samples were rinsed with PBS (2×x), and then washed with 2% BSA PBST for 30 min. After washing, samples were rinsed with distilled water (2×) before counterstaining with DAPI (23 nM) (10,236,276,001) for 10 min at room temperature. After incubation, DAPI solution was aspirated and samples were rinsed with water (2×) before coverslips were mounted to slides. Slides were imaged on an Olympus BX61 upright fluorescent microscope and the images were analyzed using ImageJ software.

### Propidium iodide stain

After initial treatment and passaging (see above), RMS cells and media supernatant were collected on day 6. After spinning down, cells were washed with PBS and fixed in 70% ethanol overnight. The next day, cells were pelleted, supernatant was aspirated, and the cells were washed (2×) with PBS. After washing with PBS, cells were pelleted and resuspended in 2% BSA PBS. Cells were counted using a hemocytometer. One million cells were aliquoted to fresh tubes and filled to 1 mL with 2% BSA PBS. Cells were incubated with RNase A (10 μg/mL) (10,109,142,001) for 30 min before addition of propidium iodide (1 μg/mL). Samples were processed on a ZE5 Cell Analyzer and analyzed with FlowJo software.

### TUNEL stain

After initial treatment and passaging (see above), RMS cells and media supernatant were collected on day 6. After spinning down, cells were washed with PBS and fixed with 1% paraformaldehyde PBS for 15 min. The ApopTag® Fluorescein Direct In Situ Apoptosis Detection Kit (S7160) was used for TUNEL staining and a modified version of their protocol was used. In short, fixed cells were collected, washed with PBS (2×), and 50,000 fixed cells (~ 20 μL) were spread evenly onto half of a coverslip. The PBS was allowed to evaporate for ~ 8 min on a 50 °C heat block to help cells stick to the coverslip. Ice-cold ethanol:acetic acid (2:1) was added to the samples for 5 min at -20 °C. After incubation, the solution was gently blotted to remove excess solution and washed with PBS (2×). Samples were incubated with equilibration buffer for 10 s at room temperature. The equilibration buffer was gently blotted and the working strength TdT enzyme was applied to the samples and incubated in a humidified chamber for 1 h at 37 °C. After incubation, the stop/wash buffer was applied to the samples for 10 min. After incubation, the samples were washed with PBS (2×) and then counterstained with DAPI (23 nM) for 10 min at room temperature. After incubation, DAPI solution was aspirated and samples were rinsed with water before coverslips were mounted to slides. Slides were imaged on an Olympus BX61 upright fluorescent microscope and the images were analyzed using ImageJ software.

### Tumor growth after SMG7 knockout

RMS cells were treated with 4-hydroxytamoxifen or ethanol as above. Forty-eight hours after treatment, cells were collected, washed with PBS (2×), counted using a hemocytometer, and resuspended in PBS at the appropriate concentration for a final injection volume of 50 μL (x cells/50 μL). Age matched male mice (2- to 3-month-old) were used for all experiments. Injection of all mice occurred at midday and in their home cage. Mice were wiped down with 70% ethanol saturated gauze to sterilize the injection site before they received a subcutaneous injection with 50 μL of cell slurry (500,000 or 10,000 cells total). At the endpoint of experiments, mice were euthanized using a CO_2_ chamber. CO_2_ flow was maintained for at least 60 s after respiratory arrest and was followed by physical death verification by bilateral thoracotomy. The tumors were collected and the mass, length and width of tumors were measured before tumor tissues were processed for downstream analysis.

### Tumor growth using a neoadjuvant approach

Clone 5 cells were collected, washed with PBS (2×), counted using a hemocytometer, and resuspended in PBS at the appropriate concentration for a final injection volume of 50 μL (200 cells/μL). Age matched male mice (2- to 3-month-old) were used for all experiments. Injection of all mice occurred at midday and in their home cage. Mice were wiped down with 70% ethanol saturated gauze to sterilize the injection site before they received a subcutaneous injection with 50 μL of cell slurry (10,000 cells total). Three days after injection of clone 5 cells, mice received intraperitoneal injections (100 μL) of tamoxifen (27 mM) or ethanol (0.857 M) corn oil on three consecutive days. At the endpoint of experiments, mice were euthanized using a CO_2_ chamber. CO_2_ flow was maintained for at least 60 s after respiratory arrest and was followed by physical death verification by bilateral thoracotomy. The tumors were collected and the mass, length and width of tumors were measured before tumor tissues were processed for downstream analysis.

### Generation of schematics

Schematics in Supplemental Fig. S1*c* and Fig. 3a were created with BioRender.com. The Albany Medical College Department of Regenerative and Cancer Cell Biology maintains a paid subscription to BioRender.com for use in journal publication.

### Equipment and settings

Western blot films were scanned using an Epson Perfection V700 PHOTO using the “Film (with Film Area Guide)” mode and at 600 dpi. Western blots were further transformed into black and white using Adobe Photoshop CS6 Extended (Image ➞ Adjustments ➞ Black & White ➞ Blue Filter). Agarose gels were imaged using BioDoc-It™ Imaging System. Agarose gel images were further processed with Adobe Photoshop CS6 Extended (Image ➞ Adjustments ➞ Levels ➞ Channel RGB Input Levels: 0–1–150). All image adjustments were applied equally to both western blots and agarose gels.

### Animal ethics and housing

All *Mus musculus* experimental protocols were approved by the Albany Medical College Institutional Animal Care and Use Committee (IACUC). Furthermore, all *Mus musculus* procedures were performed in accordance with the relevant guidelines and regulations put forth by both the ARRIVE guidelines and by the Albany Medical College Animal Resources Facility, which is licensed by the United States Department of Agriculture and the New York State Department of Health, and is accredited by the Association for Assessment and Accreditation of Laboratory Animal Care International. The ARRIVE guidelines for reporting on animal research were utilized for this report. Mice were group housed (up to five per cage) in Allentown cages under specific pathogen free conditions at the Albany Medical College Animal Resources Facility. Allentown cages had autoclaved hardwood shavings as bedding. All mice had complete and free access to water and low-fat chow. The housing rooms have a 14 h light/10 h dark cycle, an average room temperature of 72°F, and humidity range of 30–70%.

### Statistical analysis

GraphPad Prism 9 software was used for statistical analysis. Unpaired, two-tailed t-tests were utilized for the comparison of two independent groups (significance was reached if p < 0.05). One-way ANOVAs utilizing the Dunnett correction were used for western blot timeline experiments to compare control EtOH treated cells to experimental 4-OHT treated cells. For the mRNA stability assay, non-linear regression curve fitting (one phase decay) was used. Quantified data are presented as means with error bars representing the standard error of the mean. All in vitro experiments utilized at least three independent experiments. All in vivo experiments list the number of mice used in each group (see figure legends).

## Supplementary Information

Supplementary Information.

## Data Availability

The data generated and analyzed during this study are included in this published article (and its supplementary information files). All raw data pertaining to this manuscript will be provided upon request. Please refer to Y.T. for correspondence (tangy@amc.edu).

## References

[1] Bourcier K (2019). Basic knowledge in soft tissue sarcoma. Cardiovasc. Intervent. Radiol..

[2] Hayashi T (2015). Biological characterization of soft tissue sarcomas. Ann. Transl. Med..

[3] Hui JY (2016). Epidemiology and etiology of sarcomas. Surg. Clin. North Am..

[4] Rehg JE, Ward JM (2012). Morphological and immunohistochemical characterization of sarcomatous tumors in wild-type and genetically engineered mice. Vet. Pathol..

[5] Antonescu, C. R., Board, W. C. o. T. E. & Organization, W. H. *Soft Tissue and Bone Tumours*. 5 edn, 607 (International Agency for Research on Cancer: World Health Organization, 2020).

[6] Sultan I, Qaddoumi I, Yaser S, Rodriguez-Galindo C, Ferrari A (2009). Comparing adult and pediatric rhabdomyosarcoma in the surveillance, epidemiology and end results program, 1973 to 2005: an analysis of 2,600 patients. J. Clin. Oncol..

[7] Makimoto A (2022). Optimizing rhabdomyosarcoma treatment in adolescents and young adults. Cancers (Basel).

[8] Furlong MA, Mentzel T, Fanburg-Smith JC (2001). Pleomorphic rhabdomyosarcoma in adults: A clinicopathologic study of 38 cases with emphasis on morphologic variants and recent skeletal muscle-specific markers. Mod. Pathol..

[9] Cancer Genome Atlas Research, N. Comprehensive and integrated genomic characterization of adult soft tissue sarcomas. *Cell* **171**, 950–965, doi:10.1016/j.cell.2017.10.014 (2017). 10.1016/j.cell.2017.10.014 PMC5693358 29100075

[10] Shen M, Kang Y (2023). Cancer fitness genes: Emerging therapeutic targets for metastasis. Trends Cancer.

[11] Nogueira G, Fernandes R, Garcia-Moreno JF, Romao L (2021). Nonsense-mediated RNA decay and its bipolar function in cancer. Mol. Cancer..

[12] Supek F, Lehner B, Lindeboom RGH (2021). To NMD or not to NMD: Nonsense-mediated mRNA decay in cancer and other genetic diseases. Trends Genet..

[13] Lykke-Andersen S, Jensen TH (2015). Nonsense-mediated mRNA decay: An intricate machinery that shapes transcriptomes. Nat. Rev. Mol. Cell Biol..

[14] Kurosaki T, Popp MW, Maquat LE (2019). Quality and quantity control of gene expression by nonsense-mediated mRNA decay. Nat. Rev. Mol. Cell Biol..

[15] Chamieh H, Ballut L, Bonneau F, Le Hir H (2008). NMD factors UPF2 and UPF3 bridge UPF1 to the exon junction complex and stimulate its RNA helicase activity. Nat. Struct. Mol. Biol..

[16] Okada-Katsuhata Y (2012). N- and C-terminal Upf1 phosphorylations create binding platforms for SMG-6 and SMG-5:SMG-7 during NMD. Nucleic Acids Res..

[17] Boehm V (2021). SMG5-SMG7 authorize nonsense-mediated mRNA decay by enabling SMG6 endonucleolytic activity. Nat. Commun..

[18] Loh B, Jonas S, Izaurralde E (2013). The SMG5-SMG7 heterodimer directly recruits the CCR4-NOT deadenylase complex to mRNAs containing nonsense codons via interaction with POP2. Genes Dev..

[19] Unterholzner L, Izaurralde E (2004). SMG7 acts as a molecular link between mRNA surveillance and mRNA decay. Mol. Cell.

[20] Chiu SY, Serin G, Ohara O, Maquat LE (2003). Characterization of human Smg5/7a: a protein with similarities to *Caenorhabditis elegans* SMG5 and SMG7 that functions in the dephosphorylation of Upf1. RNA.

[21] Mourtada-Maarabouni M, Williams GT (2013). Growth arrest on inhibition of nonsense-mediated decay is mediated by noncoding RNA GAS5. Biomed. Res. Int..

[22] Tani H, Torimura M, Akimitsu N (2013). The RNA degradation pathway regulates the function of GAS5 a non-coding RNA in mammalian cells. PLoS ONE.

[23] Nelson JO, Moore KA, Chapin A, Hollien J, Metzstein MM (2016). Degradation of Gadd45 mRNA by nonsense-mediated decay is essential for viability. Elife.

[24] Yang X, Xie Z, Lei X, Gan R (2020). Long non-coding RNA GAS5 in human cancer. Oncol. Lett..

[25] Patel K, Murray MG, Whelan KA (2022). Roles for GADD45 in development and cancer. Adv. Exp. Med. Biol..

[26] Pastor F, Kolonias D, Giangrande PH, Gilboa E (2010). Induction of tumour immunity by targeted inhibition of nonsense-mediated mRNA decay. Nature.

[27] Monument MJ, Lessnick SL, Schiffman JD, Randall RT (2012). Microsatellite instability in sarcoma: fact or fiction?. ISRN Oncol..

[28] Yang J (2022). Therapeutic perspectives for adult soft tissue sarcoma-updates from the 2022 ASCO annual meeting. Cancer Biol. Med..

[29] Hu Z, Yau C, Ahmed AA (2017). A pan-cancer genome-wide analysis reveals tumour dependencies by induction of nonsense-mediated decay. Nat. Commun..

[30] Cowen LE, Luo H, Tang Y (2019). Characterization of SMG7 14–3-3-like domain reveals phosphoserine binding-independent regulation of p53 and UPF1. Sci. Rep..

[31] Ho K, Luo H, Zhu W, Tang Y (2021). Critical role of SMG7 in activation of the ATR-CHK1 axis in response to genotoxic stress. Sci. Rep..

[32] Cowen LE, Tang Y (2017). Identification of nonsense-mediated mRNA decay pathway as a critical regulator of p53 isoform beta. Sci. Rep..

[33] Luo H, Cowen L, Yu G, Jiang W, Tang Y (2016). SMG7 is a critical regulator of p53 stability and function in DNA damage stress response. Cell Discov..

[34] Hettmer S (2014). Anaplastic rhabdomyosarcoma in TP53 germline mutation carriers. Cancer.

[35] Mulligan LM, Matlashewski GJ, Scrable HJ, Cavenee WK (1990). Mechanisms of p53 loss in human sarcomas. Proc. Natl. Acad. Sci. USA.

[36] Thoenen E, Curl A, Iwakuma T (2019). TP53 in bone and soft tissue sarcomas. Pharmacol. Ther..

[37] Popp MW, Maquat LE (2018). Nonsense-mediated mRNA decay and cancer. Curr. Opin. Genet. Dev..

[38] Fernandes R, Nogueira G, da Costa PJ, Pinto F, Romao L (2019). Nonsense-mediated mRNA decay in development, stress and cancer. Adv. Exp. Med. Biol..

[39] Gardner LB (2010). Nonsense-mediated RNA decay regulation by cellular stress: Implications for tumorigenesis. Mol. Cancer Res..

[40] Tan K, Stupack DG, Wilkinson MF (2022). Nonsense-mediated RNA decay: An emerging modulator of malignancy. Nat. Rev. Cancer.

[41] Popp MW, Maquat LE (2015). Attenuation of nonsense-mediated mRNA decay facilitates the response to chemotherapeutics. Nat. Commun..

[42] Zhao B, Pritchard JR (2019). Evolution of the nonsense-mediated decay pathway is associated with decreased cytolytic immune infiltration. PLoS Comput. Biol..

[43] Bloethner S, Mould A, Stark M, Hayward NK (2008). Identification of ARHGEF17, DENND2D, FGFR3, and RB1 mutations in melanoma by inhibition of nonsense-mediated mRNA decay. Genes Chromosom. Cancer.

[44] Bokhari A (2018). Targeting nonsense-mediated mRNA decay in colorectal cancers with microsatellite instability. Oncogenesis.

[45] Li L (2017). The human RNA surveillance factor UPF1 modulates gastric cancer progression by targeting long non-coding RNA MALAT1. Cell Physiol. Biochem..

[46] Wang D (2011). Inhibition of nonsense-mediated RNA decay by the tumor microenvironment promotes tumorigenesis. Mol. Cell Biol..

[47] Yang L (2020). Nonsense-mediated decay factor SMG7 sensitizes cells to TNFalpha-induced apoptosis via CYLD tumor suppressor and the noncoding oncogene Pvt1. Mol. Oncol..

[48] Martin L, Gardner LB (2015). Stress-induced inhibition of nonsense-mediated RNA decay regulates intracellular cystine transport and intracellular glutathione through regulation of the cystine/glutamate exchanger SLC7A11. Oncogene.

[49] Eggermont AM, de Wilt JH, ten Hagen TL (2003). Current uses of isolated limb perfusion in the clinic and a model system for new strategies. Lancet Oncol..

[50] Wray CJ (2011). Isolated limb perfusion for unresectable extremity sarcoma: results of 2 single-institution phase 2 trials. Cancer.

[51] Horssen RV, Hagen TLMT, Eggermont AMM (2006). TNF-alpha in cancer treatment: Molecular insights, antitumor effects, and clinical utility. Oncologist.

[52] Imle R, Kommoss FKF, Banito A (2021). Preclinical in vivo modeling of pediatric sarcoma-promises and limitations. J. Clin. Med..

[53] Shum EY (2016). The antagonistic gene paralogs Upf3a and Upf3b govern nonsense-mediated RNA decay. Cell.

[54] Riehs N (2008). Arabidopsis SMG7 protein is required for exit from meiosis. J. Cell Sci..

[55] Wittkopp N (2009). Nonsense-mediated mRNA decay effectors are essential for zebrafish embryonic development and survival. Mol. Cell Biol..

[56] Chousal JN (2022). Progression of the pluripotent epiblast depends upon the NMD factor UPF2. Development.

[57] Medghalchi SM (2001). Rent1, a trans-effector of nonsense-mediated mRNA decay, is essential for mammalian embryonic viability. Hum. Mol. Genet..

[58] McIlwain DR (2010). Smg1 is required for embryogenesis and regulates diverse genes via alternative splicing coupled to nonsense-mediated mRNA decay. Proc. Natl. Acad. Sci. USA.

[59] Li T (2015). Smg6/Est1 licenses embryonic stem cell differentiation via nonsense-mediated mRNA decay. EMBO J.

[60] Skarnes WC (2011). A conditional knockout resource for the genome-wide study of mouse gene function. Nature.

[61] Testa G (2004). A reliable lacZ expression reporter cassette for multipurpose, knockout-first alleles. Genesis.

[62] Donehower LA (1992). Mice deficient for p53 are developmentally normal but susceptible to spontaneous tumours. Nature.

[63] Jacks T (1994). Tumor spectrum analysis in p53-mutant mice. Curr. Biol..

[64] Yusuf B (2013). Embryonic fibroblasts represent a connecting link between mesenchymal and embryonic stem cells. Dev. Growth Differ..

[65] Saeed H, Taipaleenmaki H, Aldahmash AM, Abdallah BM, Kassem M (2012). Mouse embryonic fibroblasts (MEF) exhibit a similar but not identical phenotype to bone marrow stromal stem cells (BMSC). Stem Cell Rev. Rep..

[66] Singhal PK (2016). Mouse embryonic fibroblasts exhibit extensive developmental and phenotypic diversity. Proc. Natl. Acad. Sci. USA.

[67] Weischenfeldt J (2008). NMD is essential for hematopoietic stem and progenitor cells and for eliminating by-products of programmed DNA rearrangements. Genes Dev..

[68] Lou CH (2014). Posttranscriptional control of the stem cell and neurogenic programs by the nonsense-mediated RNA Decay pathway. Cell Rep..

[69] Chen H (2022). UPF1 contributes to the maintenance of endometrial cancer stem cell phenotype by stabilizing LINC00963. Cell Death Dis..

[70] Bao J, Tang C, Yuan S, Porse BT, Yan W (2015). UPF2, a nonsense-mediated mRNA decay factor, is required for prepubertal Sertoli cell development and male fertility by ensuring fidelity of the transcriptome. Development.

[71] Colombo M, Karousis ED, Bourquin J, Bruggmann R, Mühlemann O (2017). Transcriptome-wide identification of NMD-targeted human mRNAs reveals extensive redundancy between SMG6- and SMG7-mediated degradation pathways. RNA.

[72] Ottens F, Boehm V, Sibley CR, Ule J, Gehring NH (2017). Transcript-specific characteristics determine the contribution of endo- and exonucleolytic decay pathways during the degradation of nonsense-mediated decay substrates. RNA.

[73] Boelz S, Neu-Yilik G, Gehring NH, Hentze MW, Kulozik AE (2006). A chemiluminescence-based reporter system to monitor nonsense-mediated mRNA decay. Biochem. Biophys. Res. Commun..

[74] Ahsaini M (2018). A rare pure embryonal rhabdomyosarcoma of the urinary bladder in an adult successfully managed with neoadjuvant chemotherapy and surgery: A case report. J. Med. Case Rep..

[75] Seitz G (2011). Treatment efficiency, outcome and surgical treatment problems in patients suffering from localized embryonal bladder/prostate rhabdomyosarcoma: A report from the cooperative soft tissue sarcoma trial CWS-96. Pediatr. Blood Cancer.

[76] Dantonello TM (2015). Tumour volume reduction after neoadjuvant chemotherapy impacts outcome in localised embryonal rhabdomyosarcoma. Pediatr. Blood Cancer.

[77] Tsumura H, Yoshida T, Saito H, Imanaka-Yoshida K, Suzuki N (2006). Cooperation of oncogenic K-ras and p53 deficiency in pleomorphic rhabdomyosarcoma development in adult mice. Oncogene.

[78] Leiner J, Le Loarer F (2020). The current landscape of rhabdomyosarcomas: An update. Virchows. Arch..

[79] Ashley CW (2021). Genetic characterisation of adult primary pleomorphic uterine rhabdomyosarcoma and comparison with uterine carcinosarcoma. Histopathology.

[80] Nakahata K (2022). K-Ras and p53 mouse model with molecular characteristics of human rhabdomyosarcoma and translational applications. Dis. Model Mech..

[81] Doyle B (2010). p53 mutation and loss have different effects on tumourigenesis in a novel mouse model of pleomorphic rhabdomyosarcoma. J. Pathol..

[82] Carvalho SD, Pissaloux D, Crombe A, Coindre JM, Le Loarer F (2019). Pleomorphic sarcomas: The State of the Art. Surg. Pathol. Clin..

[83] Huth M (2022). NMD is required for timely cell fate transitions by fine-tuning gene expression and regulating translation. Genes Dev..

[84] Lou CH (2016). Nonsense-mediated RNA decay influences human embryonic stem cell fate. Stem Cell Rep..

[85] Radzikowska J (2022). Cancer stem cell markers in Rhabdomyosarcoma in children. Diagnostics (Basel).

[86] Walter D (2011). CD133 positive embryonal rhabdomyosarcoma stem-like cell population is enriched in rhabdospheres. PLoS ONE.

[87] Wei Y (2022). Single-cell analysis and functional characterization uncover the stem cell hierarchies and developmental origins of rhabdomyosarcoma. Nat. Cancer.

[88] Gardner LB (2008). Hypoxic inhibition of nonsense-mediated RNA decay regulates gene expression and the integrated stress response. Mol. Cell Biol..

[89] Li Z, Vuong JK, Zhang M, Stork C, Zheng S (2017). Inhibition of nonsense-mediated RNA decay by ER stress. RNA.

[90] Oren YS (2014). The unfolded protein response affects readthrough of premature termination codons. EMBO Mol. Med..

[91] Kline CLB (2016). ONC201 kills solid tumor cells by triggering an integrated stress response dependent on ATF4 activation by specific eIF2α kinases. Sci. Signal..

[92] Tian X (2021). Targeting the integrated stress response in cancer therapy. Front. Pharmacol..

[93] Karam R (2015). The unfolded protein response is shaped by the NMD pathway. EMBO Rep..

[94] Shern JF (2014). Comprehensive genomic analysis of rhabdomyosarcoma reveals a landscape of alterations affecting a common genetic axis in fusion-positive and fusion-negative tumors. Cancer Discov..

[95] Casey DL (2020). Genomic determinants of clinical outcomes in rhabdomyosarcoma. Clin. Cancer Res..

[96] Chelsky ZL, Paulson VA, Chen EY (2022). Molecular analysis of 10 pleomorphic rhabdomyosarcomas reveals potential prognostic markers and druggable targets. Genes Chromosomes Cancer.

[97] Frischmeyer-Guerrerio PA (2011). Perturbation of thymocyte development in nonsense-mediated decay (NMD)-deficient mice. Proc. Natl. Acad. Sci. USA.

[98] Zetoune AB (2008). Comparison of nonsense-mediated mRNA decay efficiency in various murine tissues. BMC Genet..

[99] Huh WJ (2012). Tamoxifen induces rapid, reversible atrophy, and metaplasia in mouse stomach. Gastroenterology.

[100] Burclaff J, Osaki LH, Liu D, Goldenring JR, Mills JC (2017). Targeted apoptosis of parietal cells is insufficient to induce metaplasia in stomach. Gastroenterology.

[101] Keeley TM, Horita N, Samuelson LC (2019). Tamoxifen-induced gastric injury: Effects of dose and method of administration. Cell Mol. Gastroenterol. Hepatol..

[102] Bao X, Huang Y, Xu W, Xiong G (2020). Functions and clinical significance of UPF3a expression in human colorectal cancer. Cancer Manag. Res..

[103] Guo T (2022). RBM47 inhibits hepatocellular carcinoma progression by targeting UPF1 as a DNA/RNA regulator. Cell Death Discov..

[104] Ratnadiwakara M, Anko ML (2018). mRNA stability assay using transcription inhibition by Actinomycin D in mouse pluripotent stem cells. Bio Protoc..

