# Supplementary material for: Characterization of a rhabdomyosarcoma reveals a critical role for SMG7 in cancer cell viability and tumor growth
Source: Sci Rep. 2023 Jun 22;13:10152. doi: 10.1038/s41598-023-36568-5 (PMC10287741; doi:10.1038/s41598-023-36568-5)

# Characterization of a Rhabdomyosarcoma Reveals a Critical Role for SMG7 in Cancer Cell Viability and Tumor Growth

Alexander J. Steiner[1], Yang Zheng[1], and Yi Tang[1*]

[1]Department of Regenerative and Cancer Cell Biology, Albany Medical College, 47 New Scotland Avenue, Albany, NY 12208, United States of America

*To whom correspondence should be addressed. Tel: (518) 262-0456; Fax: (518) 262-5669; E-mail: tangy@amc.edu

Running Title: Loss of SMG7 Inhibits RMS Cell Viability & Tumor Growth

Keywords: SMG7; cancer; soft-tissue sarcoma; rhabdomyosarcoma; tumor-suppressor genes; GADD45; GAS5; cell viability; proliferation; survival; tumor growth

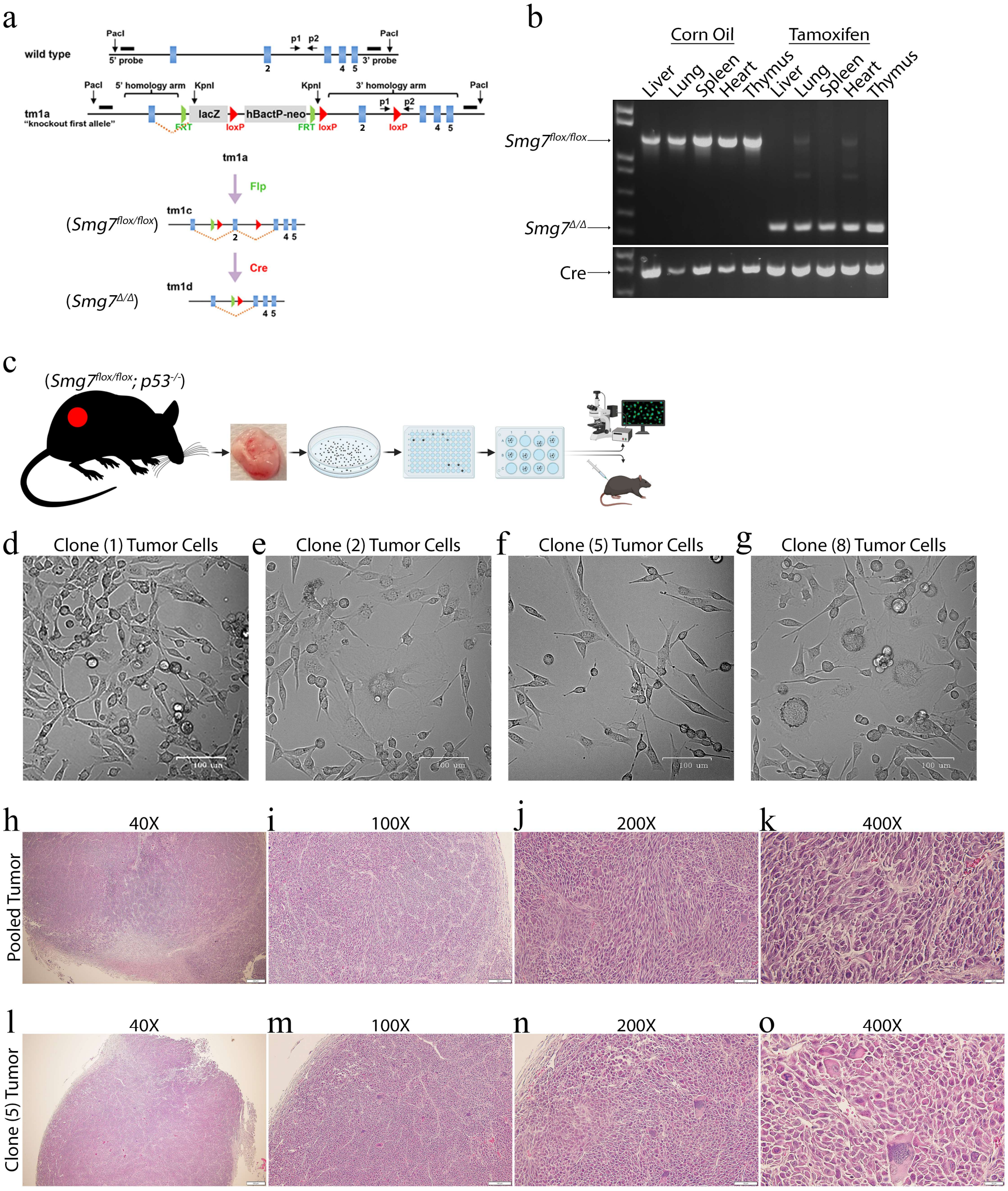


**Supplementary Figure S1: GEMM Strategy and Tumor Characterization**

**Supplementary Figure S1: GEMM Strategy and Tumor Characterization.** (a) Our *Smg7* conditional knockout mouse strategy is based on the knockout-first design.[60, 61] The second exon of *Smg7* is flanked by loxP sites (*tm1c*, referred to as *Smg7*$^{flox/flox}$). Cre-mediated deletion of the second exon of *Smg7* results in a truncated, undetectable *Smg7* product (*tm1d*, referred to as *Smg7*$^{\Delta/\Delta}$). (b) A representative genotype PCR of *Smg7*$^{flox/flox}$*; p53*$^{-/-}$ mice that were given three intraperitoneal injections of either corn oil or tamoxifen is shown. Ten days after the final injection, the mice were sacrificed and *Smg7* status was probed. (c) A 2-month-old male *Smg7*$^{flox/flox}$*; p53*$^{-/-}$ mouse presented with a subcutaneous tumor on the right-dorsal-caudal position, marked in red. The tumor cells were isolated, and aliquots of the pooled tumor cell population were frozen. The pooled tumor cell population was split into 96-well plates by limiting dilution (final concentration – 100 cells/96-well plate). Colonies were allowed to develop over a period of 2 weeks while we constantly monitored for and marked individual/multiple clone colonies. Individual clone colonies were expanded to 12-well plates, then 6 cm, and finally 10 cm plates. Pooled and clonal tumor cells were maintained for five passages before freezing and validating. To confirm the cells were of skeletal muscle lineage, the pooled and clonal tumor cells were stained with MyoD1. To validate that the tumor cells retained their tumor-initiating capacity, the pooled and clonal tumor cells were subcutaneously injected into the flanks of mice (4 mice per cell line). Tumors were collected, fixed, and then stained with H&E. The histological subtypes of tumors were characterized based on the WHO Classification of Tumours (*Soft Tissue and Bone Tumours*).[5] (d-g) Phase-contrast images (100X) of clones 1 (d), 2 (e), 5 (f), and 8 (g) (scale bars = 100 μm). (h-k) Tumors that developed after subcutaneously injecting 500,000 tumor cells were isolated and stained with H&E. Progressive magnifications are shown at 40X (h), 100X (i), 200X (j), and 400X (k). (l-o)

Tumors that developed after subcutaneously injecting 500,000 clone 5 cells were isolated and stained with H&E. Progressive magnifications are shown at 40X (l), 100X (m), 200X (n), and 400X (o).

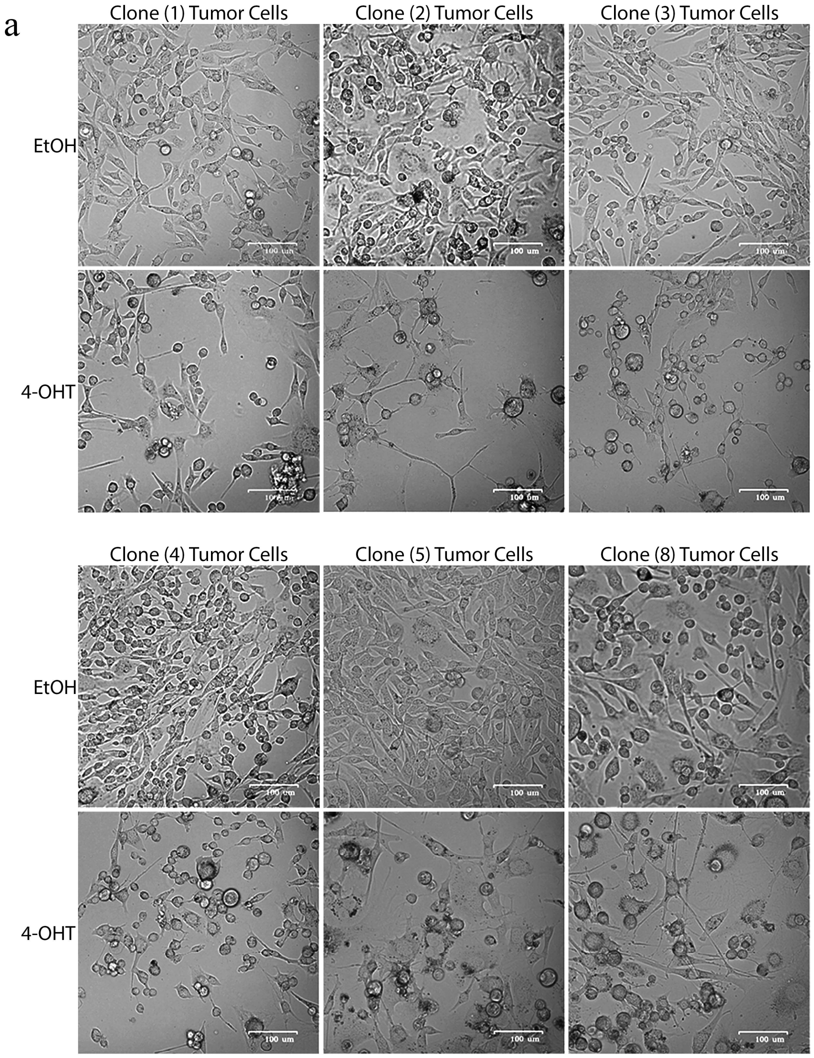


**Supplementary Figure S2: Loss of SMG7 Disrupts Normal Morphology of RMS Clones**

**Supplementary Figure S2: Loss of SMG7 Disrupts Normal Morphology of RMS Clones.**

(a) Phase-contrast images (100X) of several RMS clones seven days after EtOH or 4-OHT treatment.

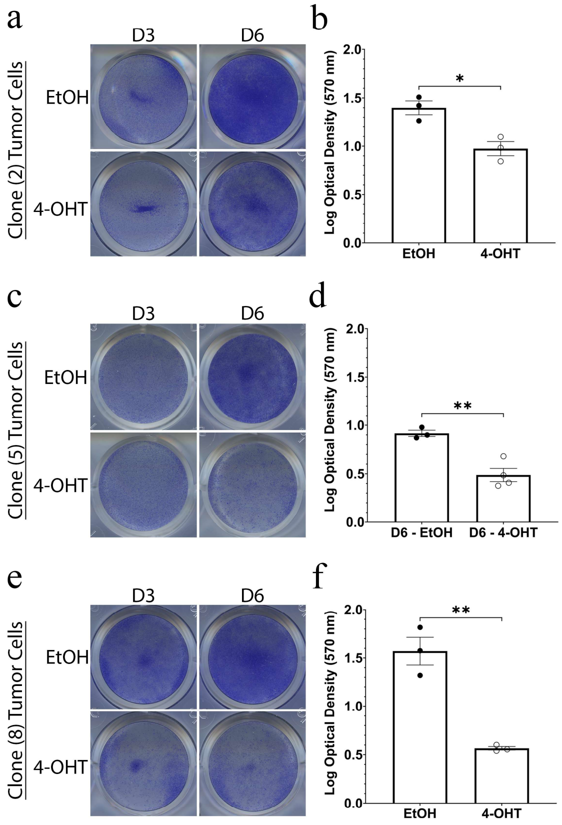


**Supplementary Figure S3: Loss of SMG7 Decreases Cell Viability of RMS Clones**

**Supplementary Figure S3: Loss of SMG7 Decreases Cell Viability of RMS Clones.** (a & b) Representative images of crystal violet staining of EtOH/4-OHT treated clone 2 cells on day 3 and day 6 are shown in (a). Optical densities of multiple, independent experiments of day 6 are quantified in (b). (c & d) Representative images of crystal violet staining of EtOH/4-OHT treated clone 5 cells on day 3 and day 6 are shown in (c). Optical densities of multiple, independent experiments of day 6 are quantified in (d). (e & f) Representative images of crystal violet staining of EtOH/4-OHT treated clone 8 cells on day 3 and day 6 are shown in (e). Optical densities of multiple, independent experiments of day 6 are quantified in (f). (Unpaired 2-tailed t-tests with means ± SEM are provided in quantifications; $N \geq 3$ ; $*P<0.05$ ; $**P<0.01$).

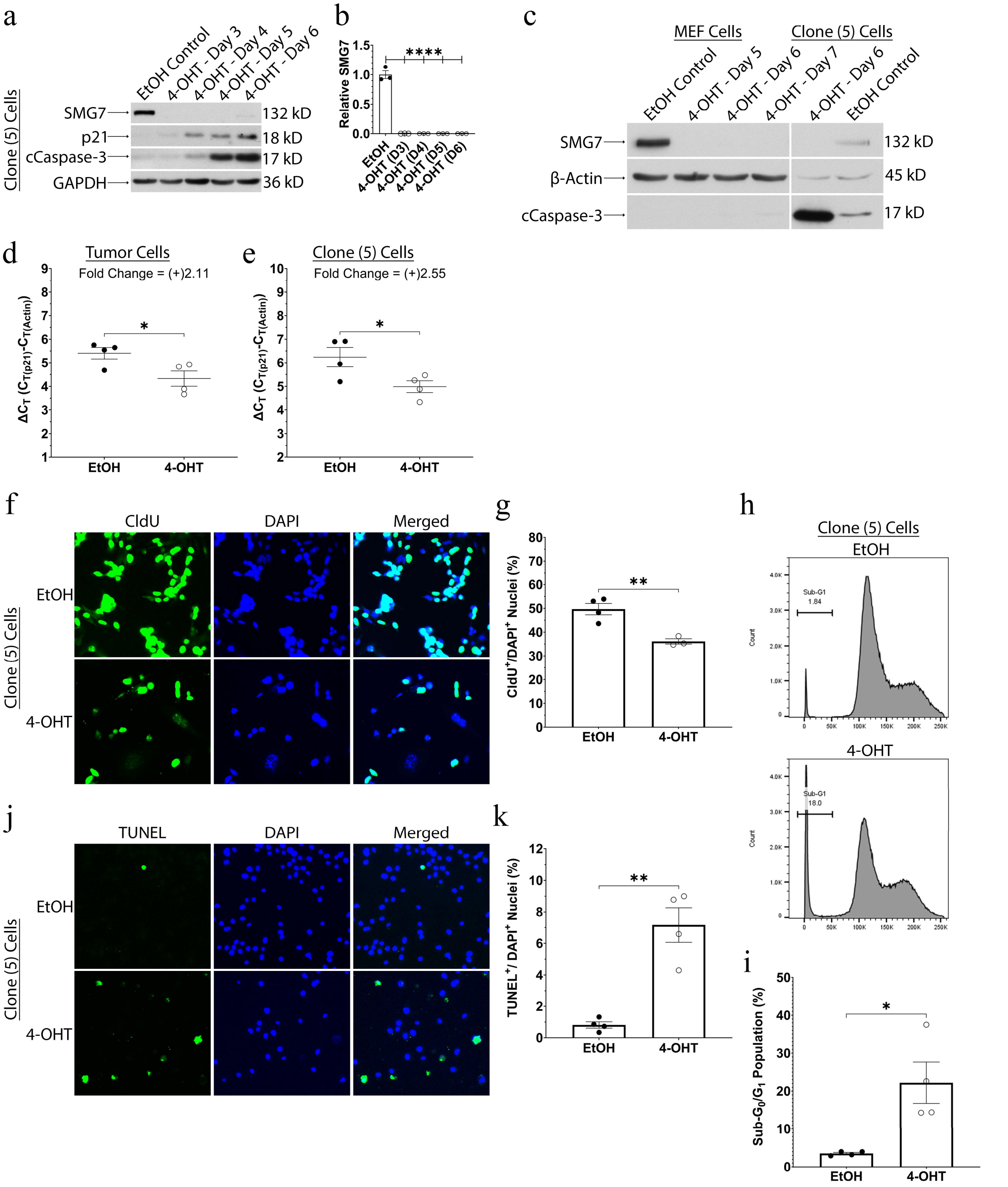


**Supplementary Figure S4: Loss of SMG7 Decreases Proliferation and Increases Apoptotic Markers in a RMS Clone but Not in MEF Cells**

**Supplementary Figure S4: Loss of SMG7 Decreases Proliferation and Increases Apoptotic Markers in a RMS Clone but Not in MEF Cells.** (a & b) Western blot of EtOH/4-OHT treated clone 5 cells showing progressive days of 4-OHT treatment. Cropped blots are shown and original blots are available at the end of the *Supplemental Figures.* Quantification of SMG7 expression, relative to EtOH controls, from multiple experiments is shown in (b). (c) Western blot of EtOH/4-OHT treated MEF cells showing progressive days of 4-OHT treatment. Day 6 EtOH/4-OHT treated clone 5 cells were run on the same blot and have the same exposures as MEF cells. They act as a reference for the cleaved caspase-3 immunoblot. Cropped blots are shown and the original blots are available at the end of the *Supplemental Figures.* (d & e) p21 qPCR analysis of day 6 EtOH/4-OHT treated tumor (d) and clone 5 (e) cells are reported as ΔCt with the relative fold changes marked above the statistics. (f & g) Representative immunofluorescence images of day 6 EtOH/4-OHT treated clone 5 cells that were processed and stained for CldU (green) and counterstained with DAPI (blue) are shown in (f). The percent of $CldU^+/DAPI^+$ cells from multiple, independent experiments are quantified in (g). (h & i) Representative flow cytometry plots of propidium iodide (PI) stained day 6 EtOH/4-OHT clone 5 tumor cells with quantification of the sub-$G_0/G_1$ peak are shown in (h). The sub-$G_0/G_1$ population of multiple, independent experiments are quantified in (i). 200,000 singlet cells were analyzed in all flow experiments. (j & k) Representative immunofluorescence images of day 6 EtOH/4-OHT treated clone 5 cells that were processed and stained for TUNEL (green) and counterstained with DAPI (blue) are shown in (j). The percent of $TUNEL^+/DAPI^+$ nuclei from multiple, independent experiments are quantified in (k). ([a & b] A one-way ANOVA utilizing the Dunnett correction with means ± SEM are provided in the quantification; [d-k] Unpaired 2-tailed t-tests with means ± SEM are provided in quantifications; N ≥ 3 ; *P<0.05 ; **P<0.01 ; ****P<0.0001 ; ns = P>0.05).

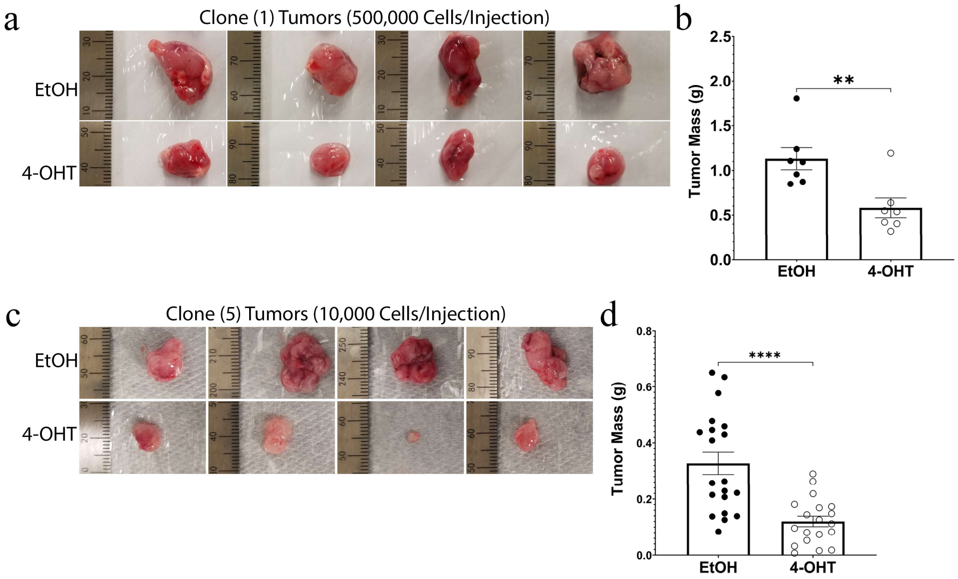


**Supplementary Figure S5: Loss of SMG7 Inhibits Tumor Growth of RMS Clones**

**Supplementary Figure S5: Loss of SMG7 Inhibits Tumor Growth of RMS Clones.** (a & b) Clone 1 cells were treated with EtOH/4-OHT for 2 days. The cells were passaged and allowed to grow for 3 more days before 500,000 cells were subcutaneously injected into the flanks of mice (EtOH treated cells injected into the right flank and 4-OHT treated cells injected into the left flank) and allowed to grow for 10 days before we sacrificed the mice and collected their tumors. Images of representative, paired tumors are shown in (a). The masses of collected tumors are quantified in (b). (c & d) Clone 5 cells were treated with EtOH/4-OHT for 2 days before 10,000 cells were subcutaneously injected into the flanks of mice (EtOH treated cells injected into the right flank and 4-OHT treated cells injected into the left flank) and allowed to grow for 14 days before we sacrificed the mice and collected their tumors. Images of representative, paired tumors are shown in (c). The masses of collected tumors are quantified in (d). (Unpaired 2-tailed t-tests with means ± SEM are provided in quantifications; (a & b) N = 7 mice; (c & d) N = 20 mice; **P<0.01 ; ****P>0.0001)

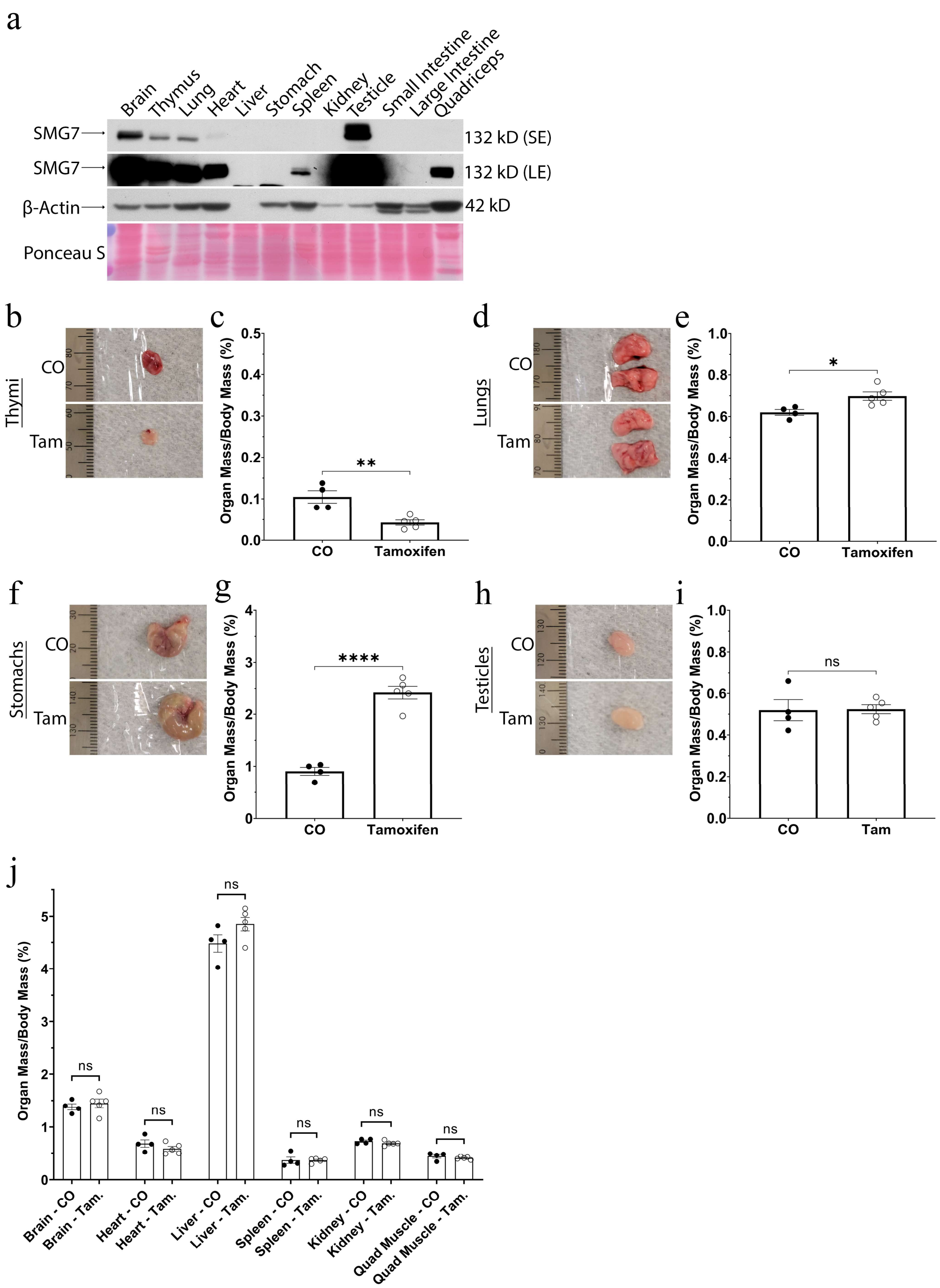


**Supplementary Figure S6: Loss of SMG7 in Adult Mice Induces Limited Gross Organ Changes**

**Supplementary Figure S6: Loss of SMG7 in Adult Mice Induces Limited Gross Organ Changes.** Adult mice injected with corn oil (CO) or Tamoxifen (Tam) on 3 consecutive days were sacrificed 14 days after the initial injections. (a) Western blot of CO injected mouse organs. A short exposure (SE) and long exposure (LE) of SMG7 immunoblot are included. Cropped blots are shown and the original blots are available at the end of the *Supplemental Figures.* (b-h) Images of representative organs from CO or Tam treated mice are shown with quantifications of relative organ mass/body mass (%) from multiple mice. Thymi are shown in (b) with quantifications in (c). Lungs are shown in (d) with quantifications in (e). Stomachs are shown in (f) with quantifications in (g). Testicles are shown in (h) with quantifications in (i). (j) Other analyzed organs from CO or Tam treated mice are accumulated into a single graph showing the relative organ mass/body mass (%). Although multiple organs are on a single graph, statistics were not used for cross comparison of different organs – Brain CO is compared to Brain Tam, not Brain CO vs Heart Tam, etcetera (j). (Unpaired 2-tailed t-tests with means ± SEM are provided in quantifications; (b-j) N = 4 CO treated mice & N = 5 Tam treated mice; *P<0.05 ; **P<0.01 ; ****P<0.0001 ; ns = P>0.05).

# Immunofluorescence Multiple Exposures - Main Figures

Fig. 1.e

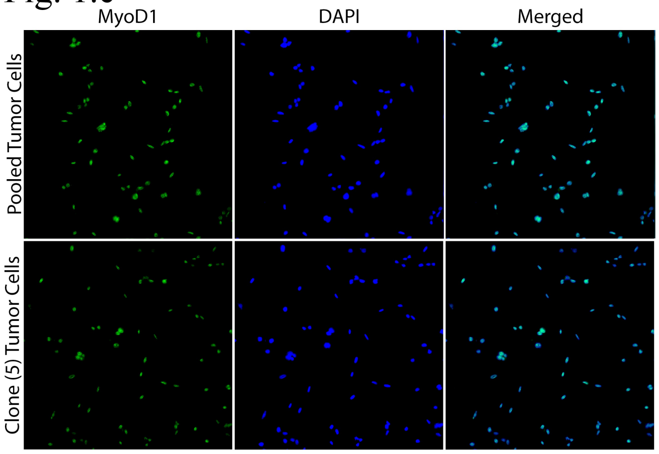


Fig. 2.i

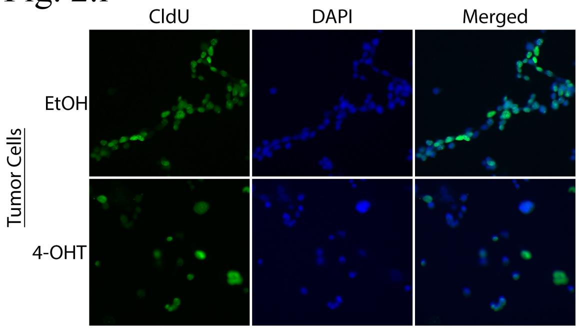


Fig. 2.m

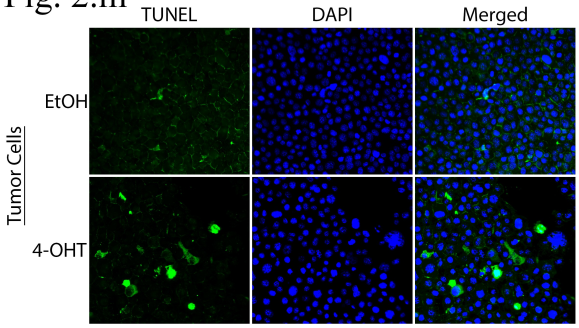

# Immunofluorescence Multiple Exposures - Supplemental Figures

Fig. S2.f

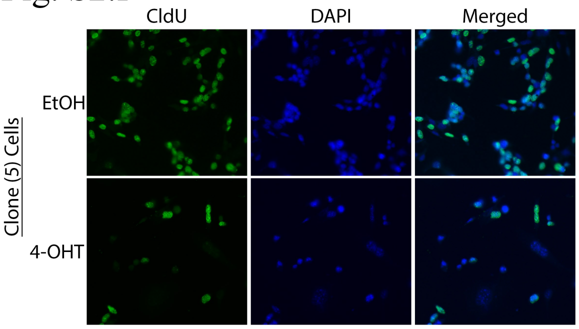


Fig. S4.j

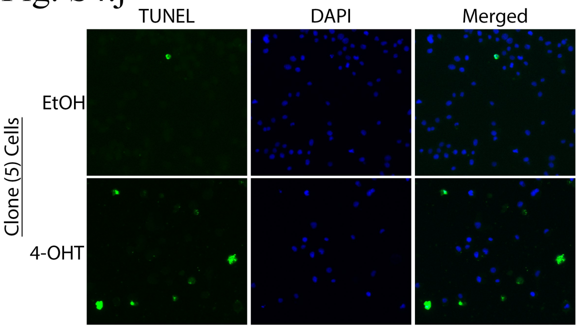

# Original Blots - Fig. 2.g - Part 1

Fig. 2.g

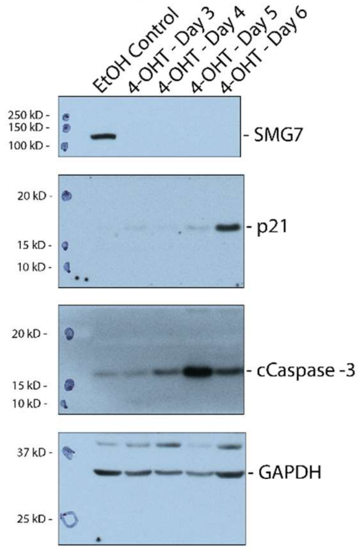


Fig. 2.g

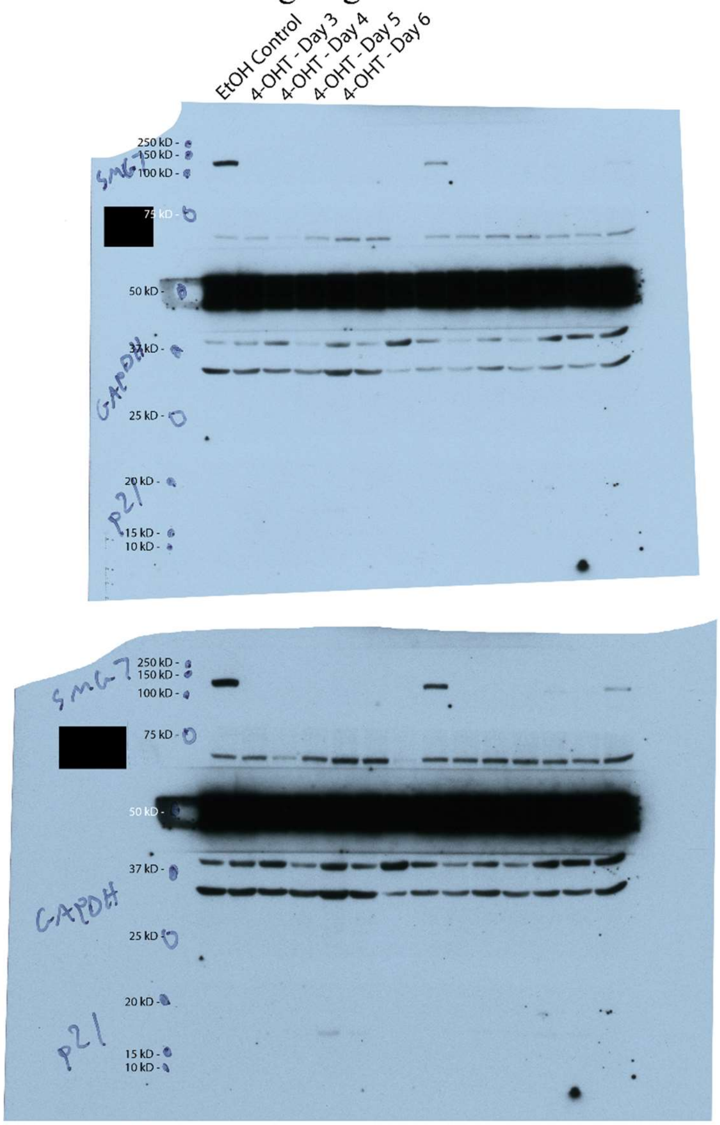

# Original Blots - Fig. 2.g - Part 2

Fig. 2.g

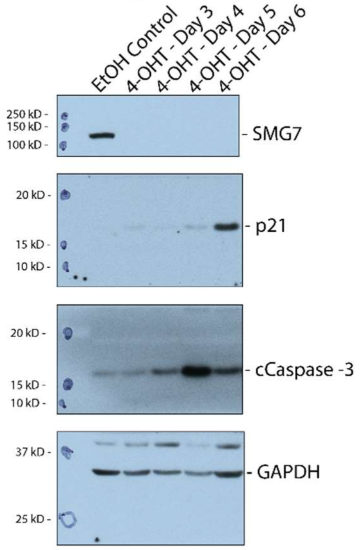


Fig. 2.g

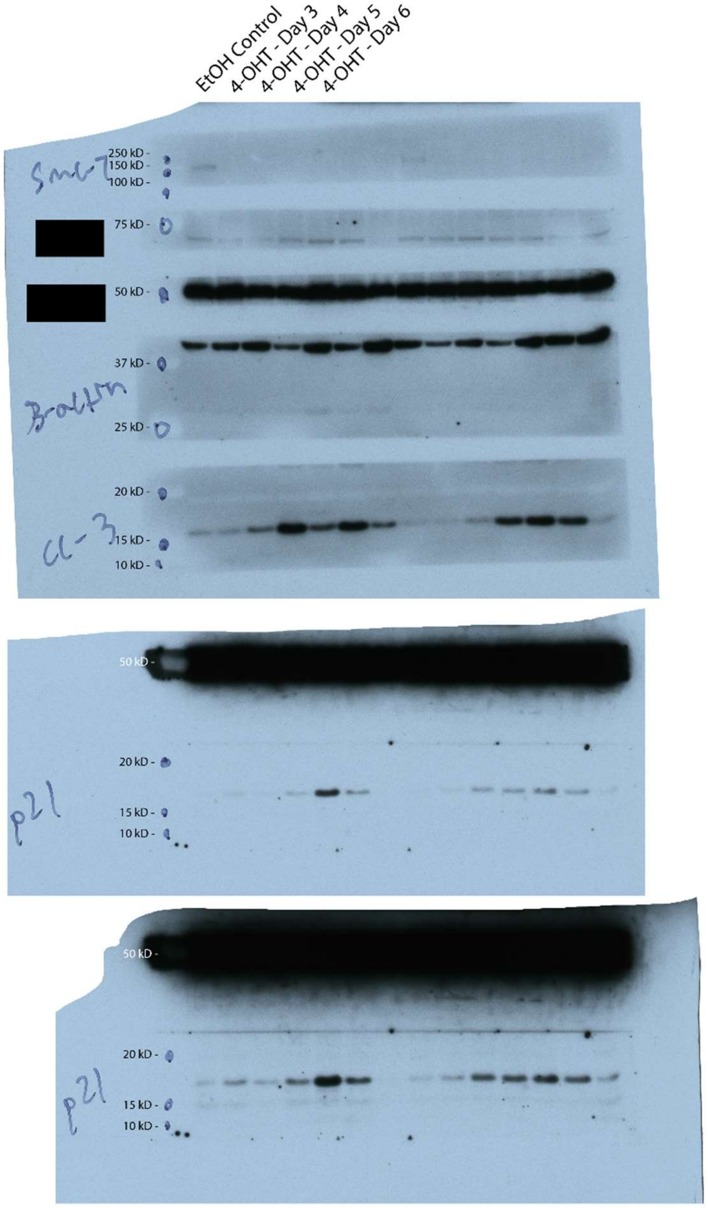

# Original Blots - Fig. 4.c - Part 1

Fig. 4.c

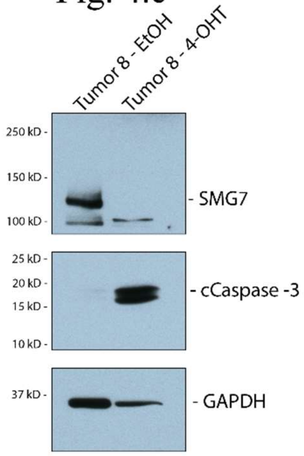


Fig. 4.c

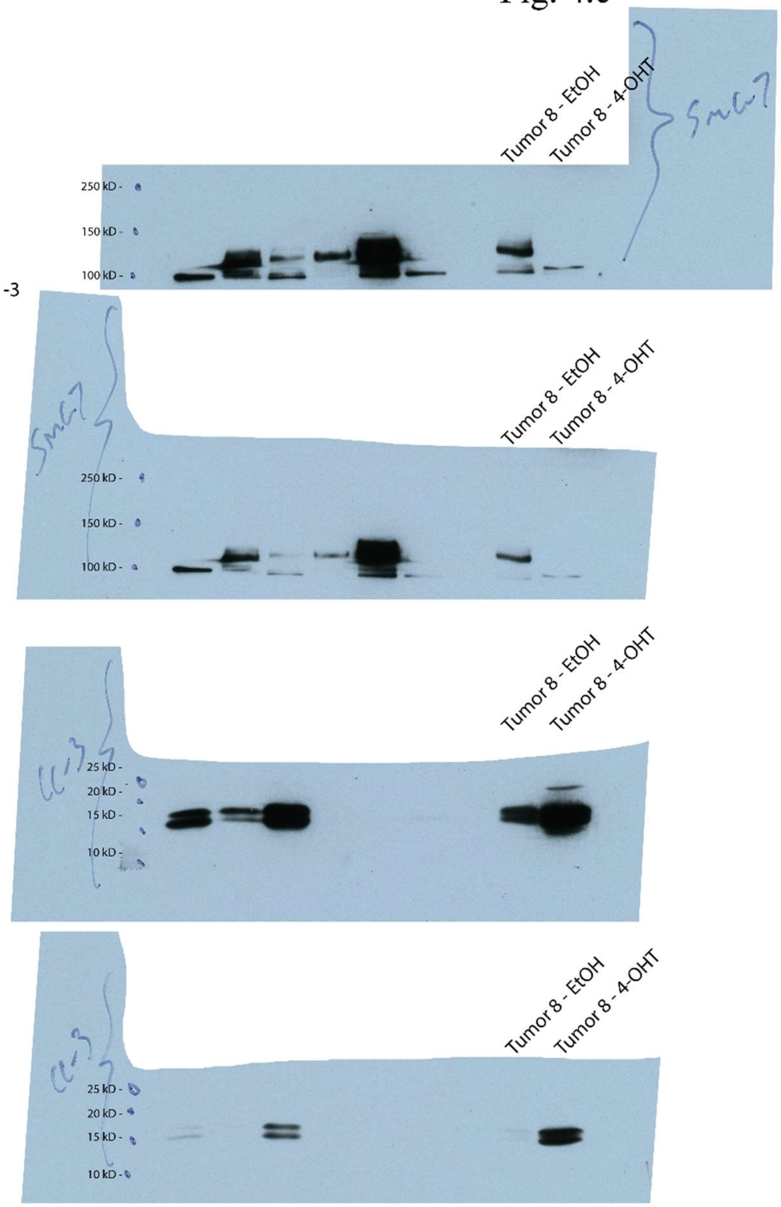

# Original Blots - Fig 4.c - Part 2

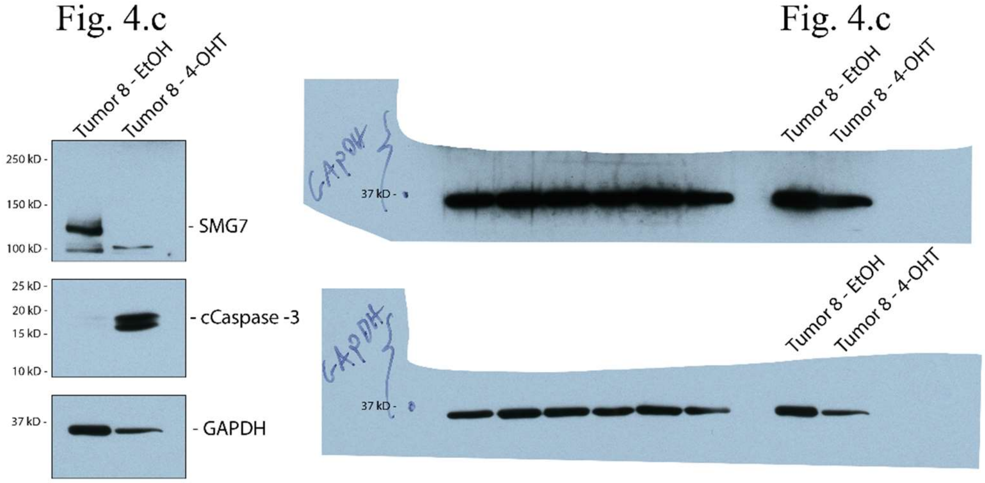

# Original Blots - Supplemental Figure S4.a - Part 1

Fig. S4.a

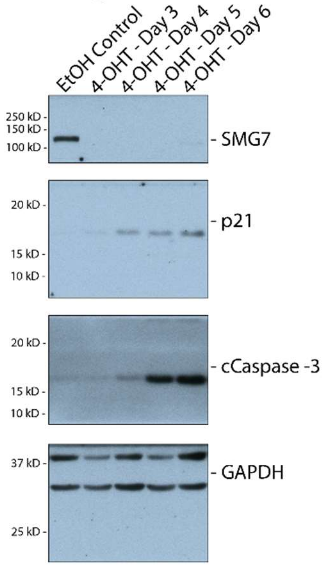


Fig. S4.a

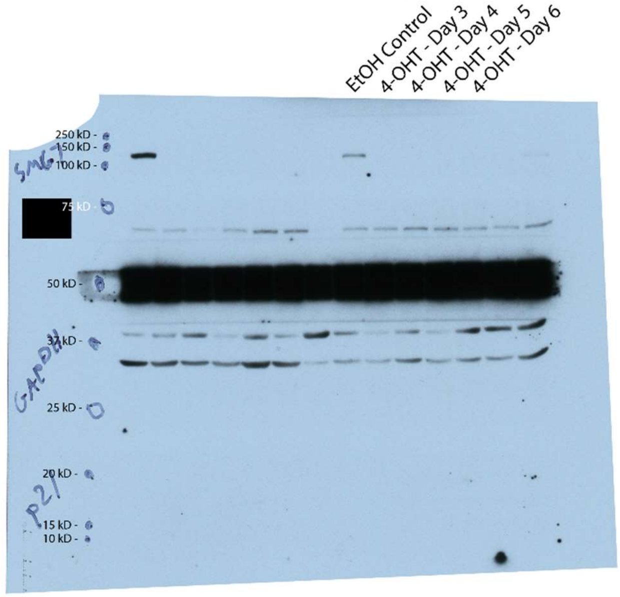


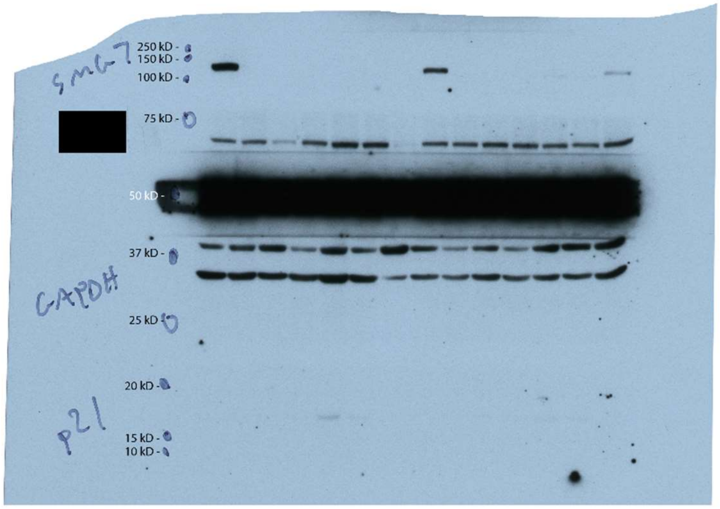

# Original Blots - Supplemental Figure S4.a - Part 2

Fig. S4.a

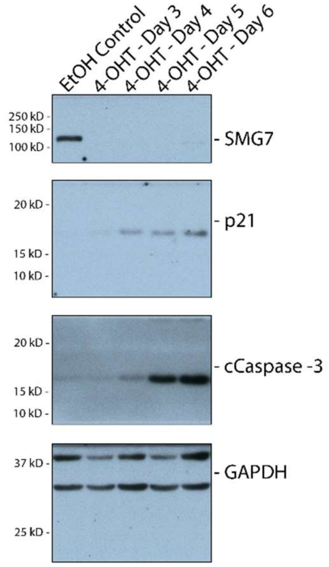


Fig. S4.a

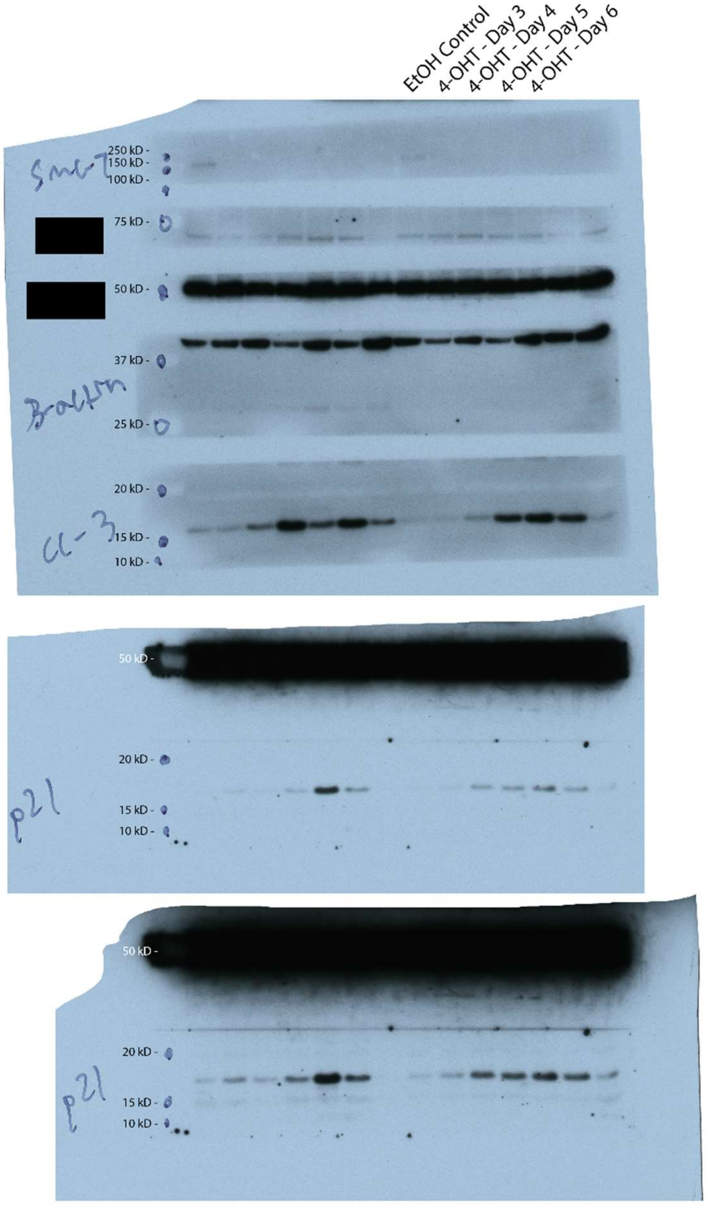

# Original Blots - Supplemental Figure S4.c - Part 1

Fig. S4.c

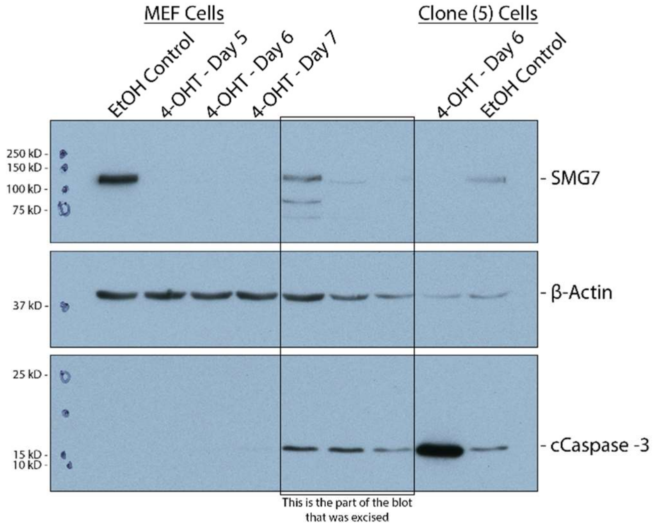


Fig. S4.c

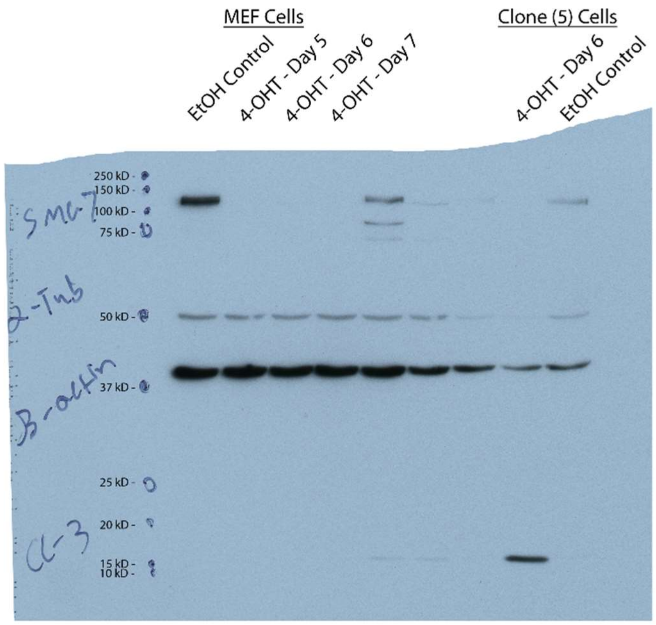

# Original Blots - Supplemental Figure S4.c - Part 2

Fig. S4.c

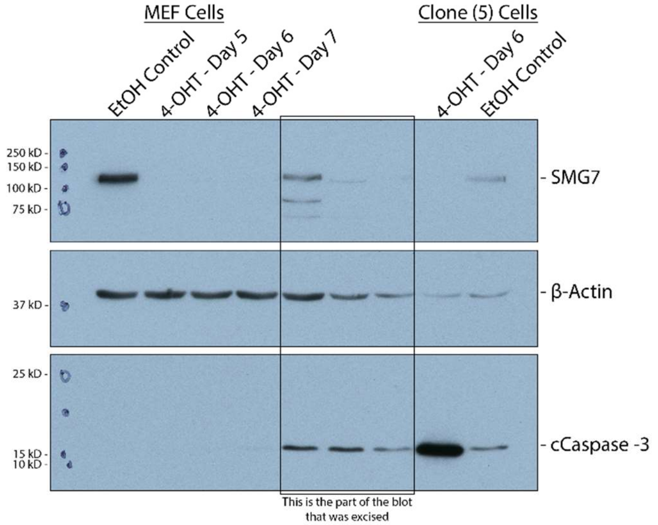


Fig. S4.c

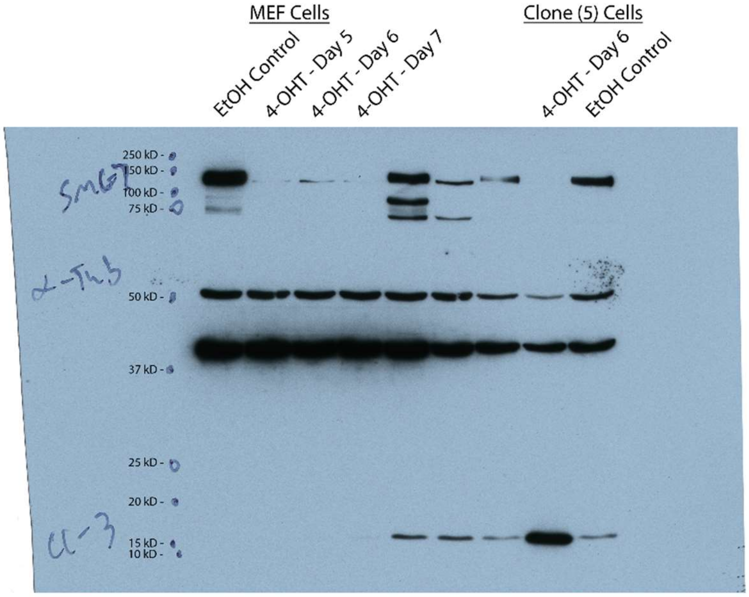

# Original Blots - Supplemental Figure S4.c - Part 3

Fig. S4.c

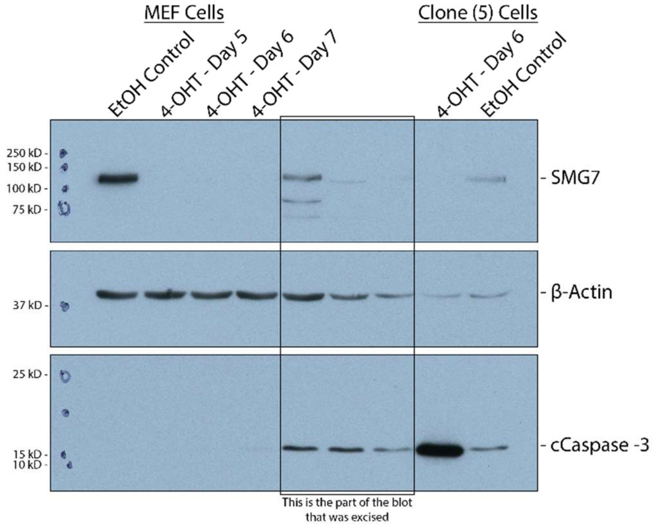


Fig. S4.c

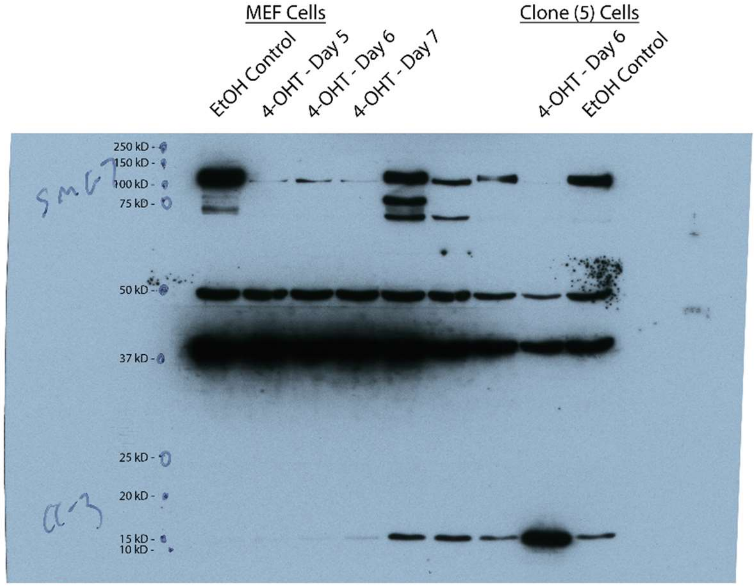

# Original Blots - Supplemental Figure S4.c - Part 4

Fig. S4.c

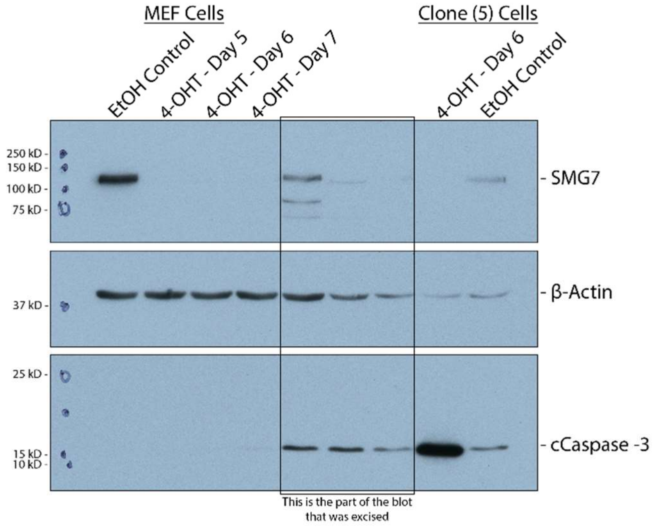


Fig. S4.c

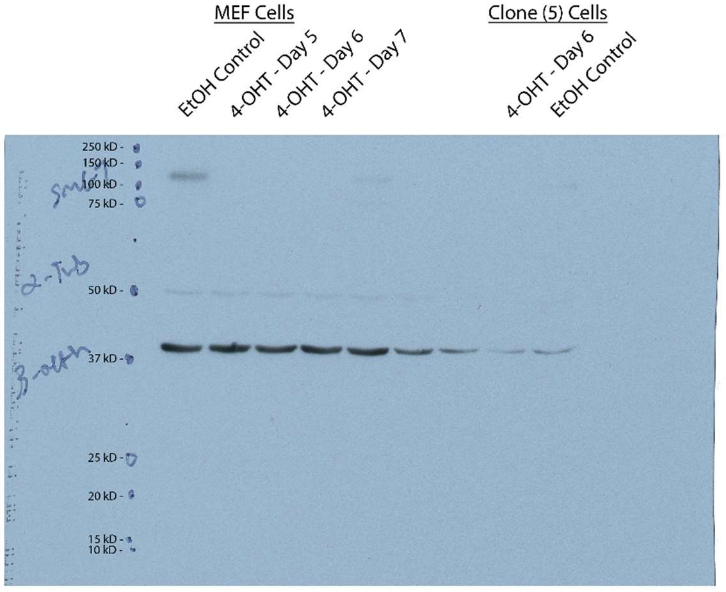

# Original Blots - Supplemental Figure S6.a - Part 1

Fig. S6.a

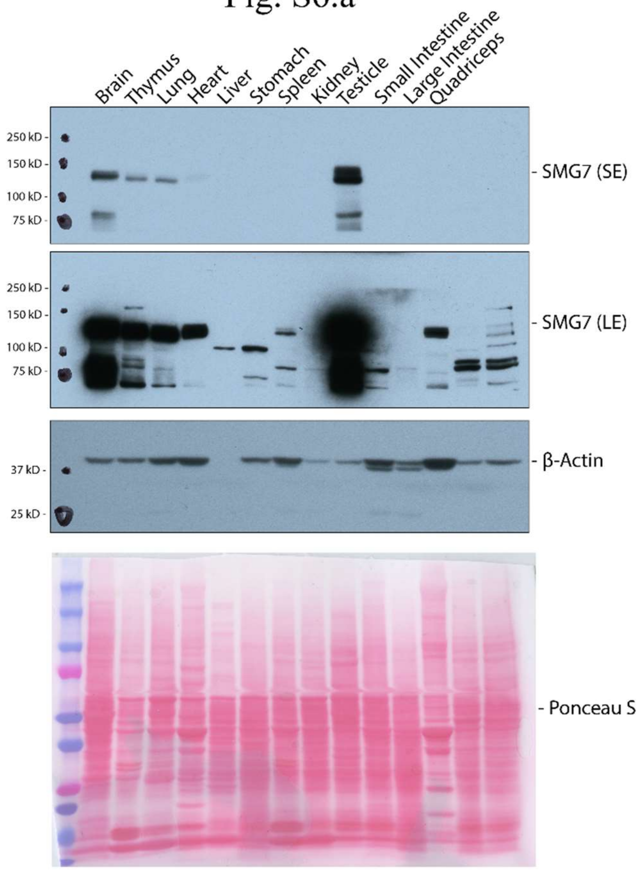

# Original Gels - Main Figures

## Fig. 3.e & 3.g

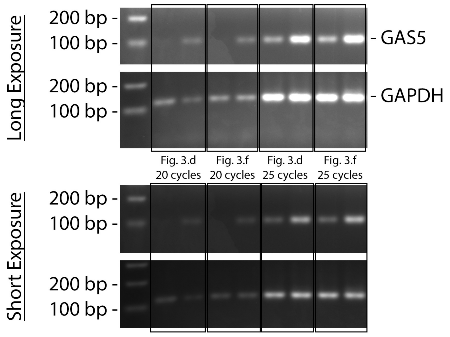


## Fig. 3.f

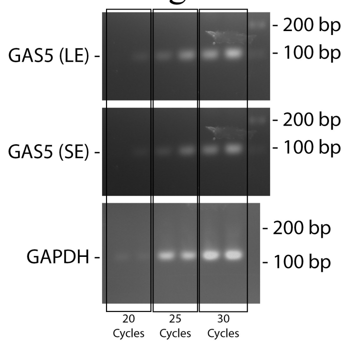


# Original Gels - Supplemental Figures

## Fig. S1.b

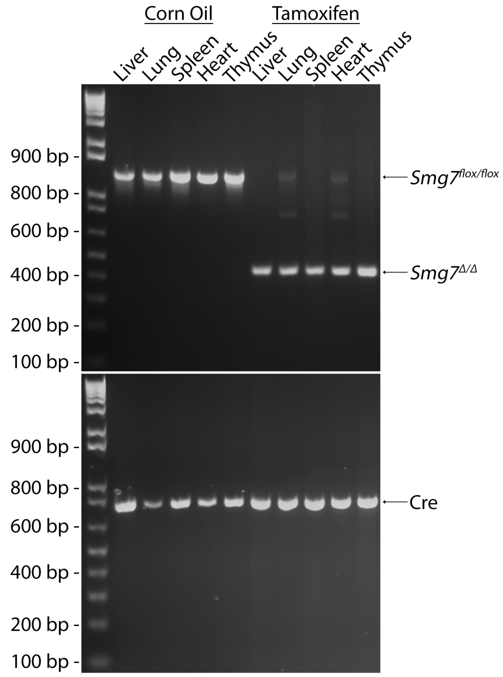

Supplement: Supplementary file 1 — Supplementary Information. [file 41598_2023_36568_MOESM1_ESM.pdf]
